# Design and Synthesis of Novel 1,2,3‐Triazole Conjugates Featuring Hydroxamic Acid and Sulfonamide Moieties: Exploring Dual Pharmacophores for Enhanced Biological Activity

**DOI:** 10.1111/cbdd.70400

**Published:** 2026-09-17

**Authors:** Dineshkumar Pagar, Ranjay Shaw, Hemant Suryavanshi, Dhanji Rajani, Sudhakar Patil

**Affiliations:** ^1^ Organic Chemistry Research Laboratory, Maharashtra Udaygiri Mahavidyalay, Swami Ramanand Teerth Marathwada University Nanded India; ^2^ Department of Chemistry GLA University Mathura Uttar Pradesh India; ^3^ Zeal College of Engineering and Research Pune India; ^4^ Microcare Laboratory Surat India

**Keywords:** 1,2,3‐Triazole, antimalarial, antimicrobial activity, antiparasitic, antitubercular, *N*‐hydroxycarboxamide, sulfonamide

## Abstract

A new series of *N*‐hydroxy‐1‐(4‐(aryl sulfonamido)phenyl)‐1*H*‐1,2,3‐triazole‐4‐carboxamide derivatives (**8a**‐**j**) were synthesized through a concise multistep route integrating three key pharmacophores: the 1,2,3‐triazole core, an aryl sulfonamide moiety, and an *N*‐hydroxycarboxamide group. Biological evaluation demonstrated promising antimicrobial potential for several derivatives. Compounds **8f**, **8g**, and **8i** exhibited notable antibacterial activity against both Gram‐positive and Gram‐negative bacteria, whereas most analogues showed weak antifungal activity. In the antimalarial assay, compound **8d** emerged as the most potent derivative (IC_50_ = 0.84 μg/mL), while compounds **8b**, **8e**, **8g**, and **8h** also displayed appreciable activity. Antitubercular evaluation identified compounds **8g** and **8i** as the most active derivatives against *Mycobacterium tuberculosis* H37Rv, with MIC values of 62.5 and 50 μg/mL, respectively. The molecular docking study against DNA gyrase (PDB ID: 7FVT) and lanosterol 14α‐demethylase (PDB ID: 5V5Z) as relevant antibacterial and antifungal targets, respectively, showed a similar trend of activity. Computational studies, including molecular docking, MM/GBSA, molecular dynamics simulations, and ADME prediction, supported the structural and pharmacokinetic characteristics of the synthesized compounds, with selected derivatives exhibiting favorable interactions with the c‐KIT kinase active site and generally acceptable drug‐like properties. Overall, these triazole–sulfonamide–N‐hydroxycarboxamide hybrids represent promising lead molecules for further structural optimization and biological investigation.

## Graphical abstract

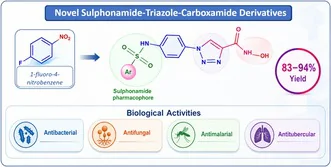

## 1 Introduction

The search for new therapeutic agents remains a fundamental pursuit in modern medicinal chemistry, driven by the growing challenges of emerging drug resistance, limited efficacy of existing drugs, and the pressing need for molecules with well‐defined mechanisms of action and improved safety profiles. Over the past three decades, triazole‐based frameworks (Kumar et al. 2025; Farwa et al. 2025; Mhetre et al. 2024; Bakale et al. 2024) and sulfonamide pharmacophores (Ghomashi, Ghomashi, Aghaei, Massah, and Massah 2023; Khan et al. 2018) have garnered significant attention as privileged scaffolds in drug discovery due to their potent biological activities, synthetic accessibility, and versatility for chemical modification, which collectively enhance pharmacokinetic and pharmacodynamic properties.

The strategic hybridization of these two moieties—triazole and sulfonamide—into a singular molecular architecture has emerged as a highly promising approach for creating novel bioactive compounds that exhibit synergistic or additive effects (Abbas et al. 2024; Albelwi et al. 2024). 1,2,3‐Triazole‐sulfonamide and related carboxamide derivatives have demonstrated broad‐spectrum biological activities, including antimicrobial, anticancer, anti‐inflammatory, enzyme inhibitory, and diagnostic applications (Abbas et al. 2024; Ghomashi, Ghomashi, Aghaei, and Massah 2023; Marinescu 2023). The introduction of electron‐withdrawing or electron‐donating substituents on the aryl sulfonamide ring has been shown to modulate potency and selectivity, making these hybrids attractive candidates for further structure–activity relationship studies and lead optimization.

Amongst the reported hybrids of 1,2,3‐triazole and sulfonamide, 4‐(1*H*‐1,2,3‐triazol‐1‐yl)benzenesulfonamides have been extensively investigated for their diverse biological activities (Albelwi et al. 2024; Singh et al. 2020; Abdelhakeem et al. 2024; Pingaew et al. 2015; Kumar et al. 2018; Tietz et al. 2025; Al‐Blewi et al. 2018; Kong et al. 2024; Yadav and Kaushik 2024, 2022). In addition, *N*‐(1*H*‐1,2,3‐triazol‐4‐yl)arylsulfonamides have also attracted considerable attention due to their notable pharmacological potential (Taheri et al. 2018; Çeşme et al. 2024, 2025; Kaushik et al. 2017; Şahin et al. 2024). Similarly, *N*‐hydroxy‐1*H*‐1,2,3‐triazole‐4‐carboxamides have also been reported to exhibit well‐defined anticancer (Wang et al. 2020; Kalinin et al. 2020) and anti‐HIV (Bai et al. 2015) activities. More recently, several structurally diverse triazole‐based hybrids, including quinoline–bis‐triazole conjugates (Sulakhe et al. 2025), triazolyl quinoline carboxylates (Bhagat et al. 2025), and isoniazid–triazole derivatives (Bakale et al. 2025), have demonstrated promising antimalarial, antimicrobial, antioxidant, and antitubercular activities. These studies highlight the versatility of the 1,2,3‐triazole scaffold; however, they are primarily based on quinoline or isoniazid pharmacophores and do not incorporate an aryl sulfonamide and/or an *N*‐hydroxycarboxamide functionality within the same molecular framework.

To the best of our knowledge, *N*‐hydroxy‐1‐(4‐(aryl sulfonamido)phenyl)‐1*H*‐1,2,3‐triazole‐4‐carboxamides have not been previously reported. In this work, a series of novel compounds were synthesized that uniquely combine the aryl sulfonamide, *N*‐hydroxycarboxamide, and 1,2,3‐triazole scaffolds within a single molecular framework, with the objective of exploring their combined influence on antimicrobial, antimalarial, and antitubercular activities. Among these derivatives, *N*‐hydroxy‐1‐(4‐(aryl sulfonamido)phenyl)‐1*H*‐1,2,3‐triazole‐4‐carboxamide analogs stand out due to the purposeful integration of essential pharmacophoric features: (1) the *N*‐hydroxycarboxamide group, which enables metal ion chelation, hydrogen bonding, and interactions with biological nucleophiles; (2) the 1,2,3‐triazole ring, which contributes to metabolic stability and acts as a rigid, bioisosteric linker; and (3) the aryl sulfonamido substituent, which is renowned for its broad biological activity and modulatory effects on the electronic and steric properties of the molecule. This rational design results in derivatives with versatile biological potentials and tunable drug‐like properties. These synthesized compounds were subsequently evaluated for several biological activities, including antibacterial, antifungal, antimalarial, and antitubercular effects. Additionally, the potential kinase‐binding capability of the compounds was evaluated against a Human c‐KIT kinase.

## 2 Result and Discussion

### 2.1 Synthesis

**SCHEME 1:**
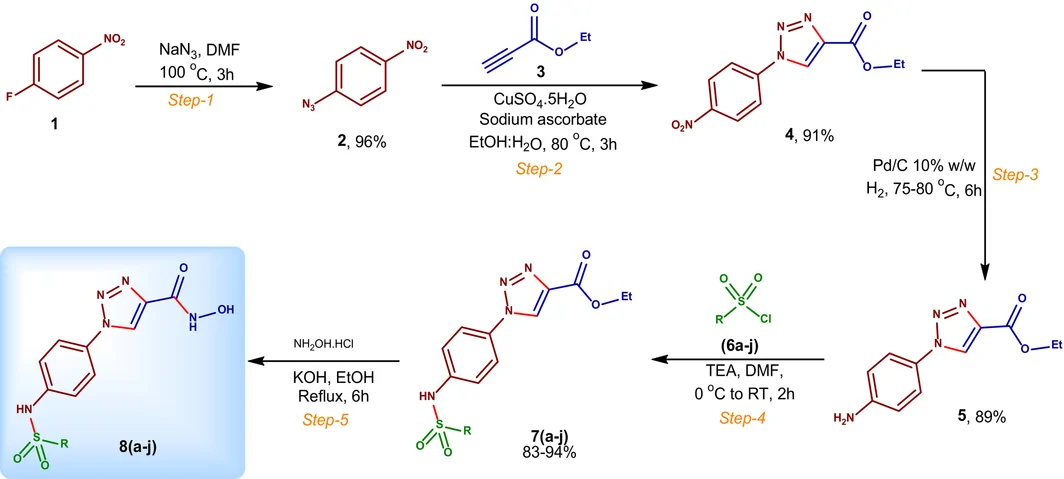
Synthesis of *N*‐hydroxy‐1‐(4‐(aryl sulfonamido)phenyl)‐1*H*‐1,2,3‐triazole‐4‐carboxamide.

A multistep synthetic strategy was adopted to obtain the *N*‐hydroxy‐1‐(4‐(aryl sulfonamido)phenyl)‐1*H*‐1,2,3‐triazole‐4‐carboxamide derivatives (Scheme 1). The sequence commenced with the nucleophilic azidation of 1‐fluoro‐4‐nitrobenzene (**1**) using sodium azide in DMF, affording the corresponding azide (**2**). The azide intermediate (**2**) was then subjected to a copper‐catalyzed azide–alkyne cycloaddition (CuAAC) with ethyl propiolate (**3**), producing the 1,4‐disubstituted 1,2,3‐triazole scaffold (**4**) in excellent yield. Subsequent hydrogenation of the nitro functionality in compound **4** was carried out using 10% Pd/C under hydrogen atmosphere at 75°C–80°C, yielding the amine intermediate **5** (ethyl 1‐(4‐aminophenyl)‐1H‐1,2,3‐triazole‐4‐carboxylate). The aminated compound **5** was then reacted with various substituted sulfonyl chlorides (**6a–j**) in the presence of triethylamine in DMF to afford the sulfonamide‐linked triazoles (**7a–j**) in good to excellent yields. Finally, refluxing compounds **7a–j** with hydroxyl amine hydrochloride in an alkaline alcoholic medium furnished the desired *N*‐hydroxy‐triazole carboxamide derivatives **8a–j**. The isolated yields of products **8a–j** are summarized in Table 1.

**TABLE 1:** Different sulfonamide‐linked *N*‐hydroxy‐triazole carboxamides **(8a–j)**.

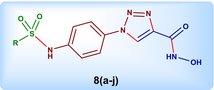			
Product	*R*	Yield (%)^a^	M.P. (°C)
**8a**	4‐Me.C_6_H_4_	81	168–170
**8b**	3‐Me.C_6_H_4_	75	153–155
**8c**	4‐F.C_6_H_4_	79	181–183
**8d**	3‐F.C_6_H_4_	76	170–172
**8e**	4‐Cl.C_6_H_4_	83	191–193
**8f**	3‐Cl.C_6_H_4_	78	180–182
**8g**	4‐OMe.C_6_H_4_	85	173–175
**8h**	3,4‐Cl.C_6_H_3_	76	214–216
**8i**	2,5‐Cl.C_6_H_3_	72	190–192
**8j**	3,5‐Cl.C_6_H_3_	75	205–207

^a^ Isolated yield.

All sulfonamide‐triazole carboxamides **(8a–j)** were obtained in excellent yields (72%–85%) as crystalline solids and were characterized by IR, NMR, and HRMS analyses. The IR spectra displayed strong absorptions at 1640–1646 cm^−1^, characteristic of the amide carbonyl group, along with broad N‐H stretching bands at 3136–3169 cm^−1^, O‐H bending vibration near 1223–1249 cm^−1^ and two strong absorption bands for sulfonamide carbonyl: an asymmetric stretch at 1330–1356 cm^−1^ and a symmetric stretch at 1150–1181 cm^−1^. ^1^H‐NMR spectra consistently showed the *N*‐hydroxycarboxamide NH peaks at δ 9.11–9.18 ppm merged with the triazole C‐H peak, and the OH peak at 11.39–11.46 ppm (bs, 1H). The sulfonamide NH appeared downfield between δ 9.34–10.86 ppm. In the ^13^C‐NMR spectra, the triazole and aromatic carbons appeared between δ 120–125 ppm, and the amide carbonyl carbon was observed at δ 157–159 ppm. HRMS analysis supported the proposed structures, with observed [M + H]^+^ peaks in excellent agreement with the calculated value. Overall, the combined spectroscopic data unambiguously confirmed the successful synthesis of *N*‐hydroxy‐1‐(4‐(aryl sulfonamido)phenyl)‐1*H*‐1,2,3‐triazole‐4‐carboxamide (**8a–j**), with substituent‐dependent variations in chemical shifts, providing further structural validation.

A series of *N*‐(4‐(1*H*‐1,2,3‐triazolyl)phenyl)arylsulfonamides (**7**) were synthesized through well‐established sequential transformations. Initially, aryl azide intermediate (**2**) was prepared via nucleophilic aromatic substitution of a nitro‐substituted aryl fluoride with azide ion through a Meisenheimer intermediate. The resulting aryl azide underwent a copper (I)‐catalyzed azide–alkyne cycloaddition (CuAAC) with alkyne (**3**), generating the 1,4‐disubstituted 1,2,3‐triazole (**4**) regioselectively via a copper acetylide intermediate. Subsequent Pd/C‐catalyzed hydrogenation reduced the nitro group to the corresponding aniline (**5**). Sulfonamide derivatives (**7**) were obtained by nucleophilic substitution of various aryl sulfonyl chlorides with an aniline intermediate in the presence of triethylamine. Finally, the ester group of compounds **7** was converted to the corresponding *N*‐hydroxyamide (**8**) via nucleophilic acyl substitution with hydroxylamine.

### 2.2 Biological Activity

#### 2.2.1 Antibacterial Activity (In Vitro and Docking Study)

The synthesized 1,2,3‐triazole‐4‐carboxamide (**8a–j**) were assessed for their antibacterial activity against a set of four bacterial strains. This set consisted of two Gram‐negative bacteria, *Escherichia coli* (MTCC 443) and *Pseudomonas aeruginosa* (MTCC 1688), and two Gram‐positive bacteria, *Staphylococcus aureus* (MTCC 96) and *Streptococcus pyogenes* (MTCC 442) (Table 2).

**TABLE 2:** In vitro anti‐bacterial activity of compounds (**8a–j**).

Bacterial strain	*E. coli* (MTCC 443)	*P. aeruginosa* (MTCC 1688)	*S. aureus* (MTCC 96)	*S. pyogenus* (MTCC 442)
Compound				
**5**	250	100	125	125
**8a**	100	62.5	250	250
**8b**	250	100	100	100
**8c**	100	125	250	250
**8d**	250	250	125	100
**8e**	125	100	250	250
**8f**	100	62.5	100	62.5
**8g**	100	50	100	62.5
**8h**	125	250	250	250
**8i**	100	250	125	250
**8j**	100	50	125	125
Chloramphenicol	50	50	50	50
Ampicillin	30	—	40	25
Ciprofloxacin	25	25	50	50
Norfloxacin	10	10	10	10

**FIGURE 1:**
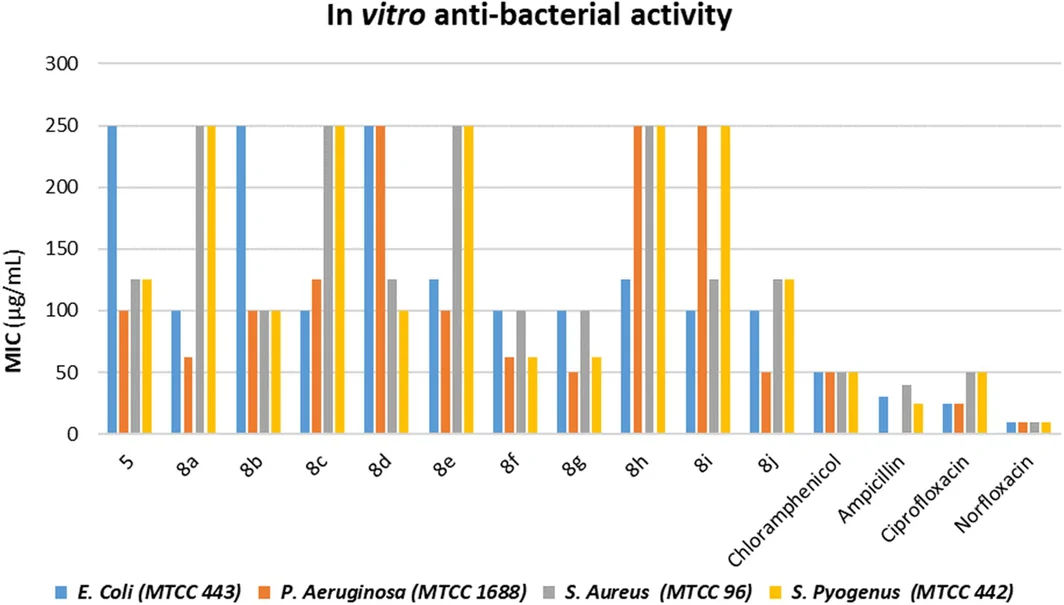
In vitro antibacterial activity of compounds (**8a–j**).

Among the synthesized derivatives, compounds **8a, 8c, 8f, 8g, 8i**, and **8j** exhibited notable antibacterial activity against *E. coli* , with minimum inhibitory concentration (MIC) values of 100 μg/mL, although their efficacy was lower than that of the reference drug chloramphenicol (MIC = 50 μg/mL) (Figure 1). Notably, compounds **8g** (*4OMe substituted*) and **8j** (*3,5‐Cl substituted*) displayed the highest activity against *P. aeruginosa* (MIC = 50 μg/mL), whereas **8a** (*4Me substituted*) and **8f** (*3Cl substituted*) showed moderate inhibition with MIC values of 62.5 μg/mL. Similarly, compounds **8b**, **8f**, and **8g** exhibited moderate antibacterial activity against *S. aureus* (MIC = 100 μg/mL), while **8f** and **8g** were also active against *S. pyogenes* . No distinct trend was observed in the selectivity of the compounds toward Gram‐negative or Gram‐positive bacterial strains. Overall, compounds **8f**, **8g**, and **8j** emerged as the most promising candidates, highlighting *meta‐chloro* and *para‐methoxy* substituents in carboxamides demonstrating significant antibacterial potential against both Gram‐negative ( *P. aeruginosa* ) and Gram‐positive ( *S. pyogenes* ) bacteria. These observations suggest that both electron‐donating (CH₃ and OCH₃) and electron‐withdrawing (Cl) substituents can enhance antibacterial activity, although their influence appears to depend strongly on the substitution pattern. The comparable activities of *meta*‐chloro and *para*‐methoxy analogues indicate that antibacterial potency is governed by a delicate balance of electronic effects, lipophilicity, steric factors, and hydrogen‐bonding capability rather than by the electronic nature of the substituent alone. A comparison with the antibacterial activity of compound **5** revealed that the incorporation of certain sulfonamide and *N*‐hydroxycarboxamide functionalities likely contributed to the enhanced antibacterial potency observed in these derivatives.

In concurrence with the antibacterial data, the molecular docking results indicate that compounds **8a**‐**8j** possess favorable binding within the active site of DNA gyrase of *Staphylococcus aureus* (PDB ID: 7FVT, Resolution = 2.08 Å), although the docking score does not show a direct one‐to‐one correlation with the observed MIC values (Cumming et al. 2023). The docking scores of the synthesized compounds ranged from −5.08 to −5.94 kcal/mol (Table 3). Among the series, **8c** exhibited the most effective docking score (−5.94 kcal/mol), followed by **8j** (−5.73), **8e** (−5.65), **8g** (−5.49), **8h** (−5.40), **8a** (−5.34), **8f** (−5.26), **8d** (−5.21), **8i** (−5.11) and **8b** (−5.08 kcal/mol). The reference compounds showed variable docking scores, with cephalexin (−6.017 kcal/mol), amoxicillin (−5.953 kcal/mol), and cefixime (−5.74 kcal/mol) comparable to the best‐performing synthesized compounds.

**TABLE 3:** Docking properties of ligands **8a–j** against the active site of DNA gyrase.

Title	Docking score	Glide energy	Lipophilic cont.	H‐bond cont.	Electro‐static cont.	XP sitemap cont.	XP LowMW cont.	XP rotational penalty	XP expose penalty	XP pose rank	Rotatable bonds
**8c**	−5.94	−72.21	−3.52	−1.29	−0.88	−0.37	−0.24	0.35	0.00	1	7
**8j**	−5.73	−44.43	−3.75	−0.80	−2.04	−0.18	−0.07	0.20	0.27	1	7
**8e**	−5.65	−47.77	−3.90	−1.13	−0.85	0.00	−0.19	0.32	0.10	1	7
**8g**	−5.49	−57.20	−4.06	−0.83	−0.73	0.00	−0.20	0.33	0.00	1	8
**8h**	−5.40	−55.47	−4.32	−0.83	−0.60	0.00	−0.07	0.28	0.14	1	7
**8a**	−5.34	−54.38	−3.95	−0.83	−0.66	0.00	−0.26	0.36	0.00	1	7
**8f**	−5.26	−59.05	−3.55	−0.94	−2.11	−0.14	−0.19	0.23	0.80	1	7
**8d**	−5.21	−60.75	−3.40	−0.83	−0.69	−0.40	−0.24	0.35	0.00	1	7
**8i**	−5.11	−62.26	−3.83	−0.83	−0.66	0.00	−0.07	0.28	0.00	1	7
**8b**	−5.08	−57.37	−3.68	−0.83	−0.68	0.00	−0.26	0.36	0.00	1	7
Co‐crys. Lig.	−9.97	−52.88	−7.15	−0.25	−0.97	−0.40	−0.02	0.22	0.10	1	6
Cephalexin	−6.02	−58.18	−3.99	−1.50	−0.46	0.00	−0.34	0.27	0.00	1	6
Amoxicillin	−5.95	−61.62	−2.93	−1.89	−1.06	−0.01	−0.28	0.22	0.00	1	7
Cefixime	−5.74	−58.20	−3.33	−1.69	−1.10	0.00	0.00	0.38	0.00	1	11

**FIGURE 2:**
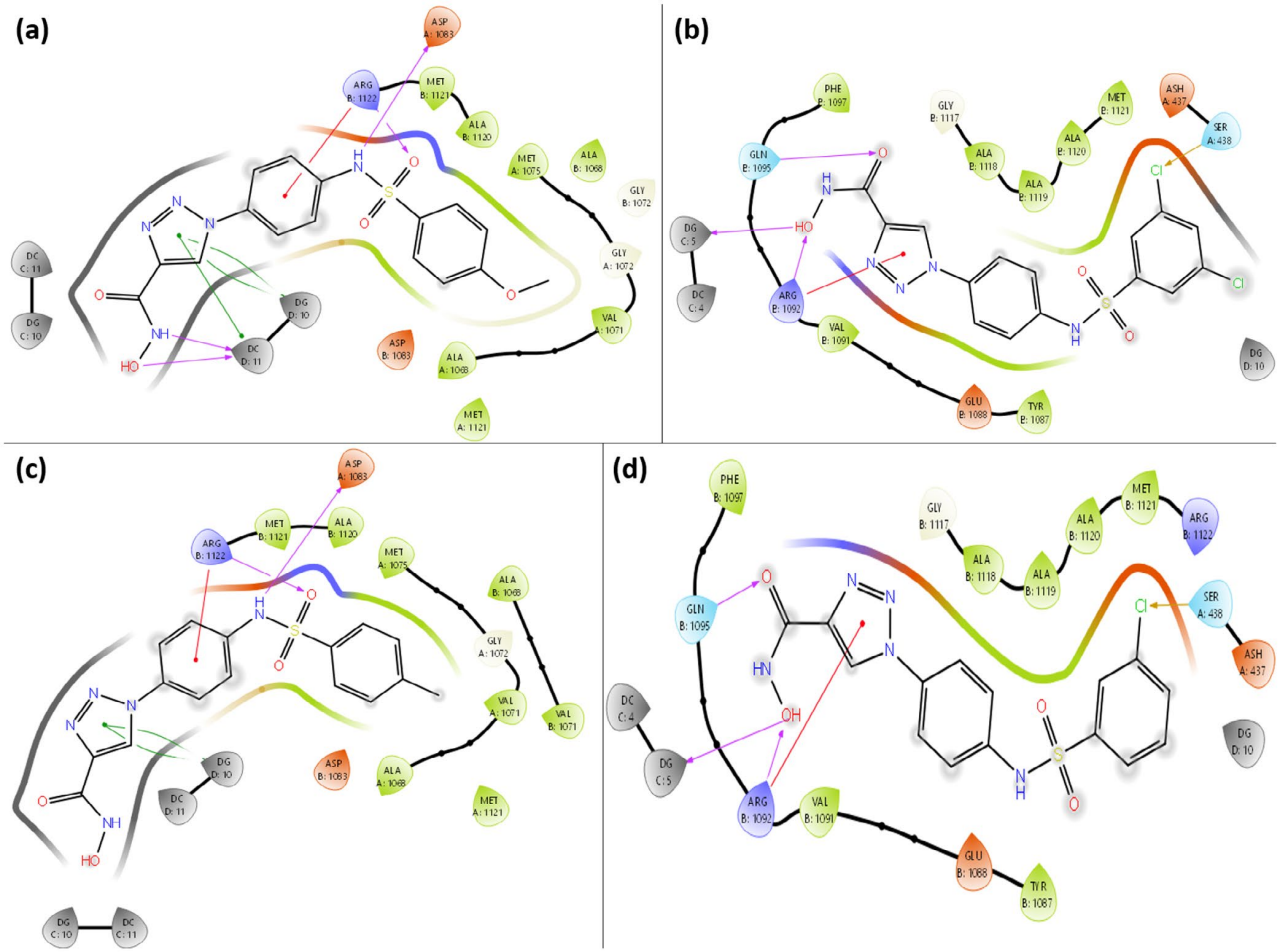
2D molecular docking interaction diagrams of the synthesized compounds (a) **8g**, (b) **8j**, (c) **8a**, and (d) **8f** within the active site of DNA gyrase, illustrating the key ligand‐residue interactions involved in their predicted binding. The corresponding 2D docking interaction diagrams of the remaining synthesized compounds and standard drugs were provided in the Figures S1 and S2.

The docking poses and decomposition of docking score indicate that the compounds are primarily stabilized through hydrophobic contacts, along with a combination of hydrogen‐bonding and π‐related interactions. Although there are small energetic penalties due to rotational restriction and unfavorable exposure of ligand groups to solvent, XP Pose rank 1 indicates a well‐fitting docking pose of the ligands in the active site. In particular, the sulfonamide moiety of several derivatives formed *H*‐bonding interactions with ASP1063 and ARG1122 residues, while the aromatic and heterocyclic regions occupy hydrophobic portions of the binding pocket (Figure 2a–d, Figures S1 and S2). Whereas, the *N*‐hydroxyl amide showed *H*‐bonding interactions with ARG1092 and GLN1095 residues. Additional interactions with DNA bases such as deoxyguanosine (DG 5 or 10) and deoxycytidine (DC 4 or 11) are observed in several complexes, suggesting that the heterocyclic portion may contribute to the stabilization of the ligand within the protein‐DNA environment. The docking poses of **8g**, despite having a score of −5.49 kcal/mol rather than the best score of the series, display a well‐oriented interaction network involving the sulfonamide group and surrounding hydrophobic residues. This is consistent with its excellent antibacterial profile, with MIC values of 100, 50, 100 and 62.5 μg/mL against *E. coli* , *P. aeruginosa* , *S. aureus* , and *S. pyogenes* , respectively. Similarly, **8f** (−5.26 kcal/mol) showed broad‐spectrum antibacterial activity (MIC 100, 62.5, 100 and 62.5 μg/mL), while **8j** (−5.73 kcal/mol) showed particularly strong activity against *P. aeruginosa* (MIC 50 μg/mL). Interestingly, **8c** had the best docking score but comparatively weaker antibacterial activity, demonstrating that docking score alone cannot predict biological potency. Overall, the combined results identify **8g** as the most promising antibacterial lead, with **8f** and **8j** also showing moderate activity and meaningful target interactions.

#### 2.2.2 Antifungal Activity (In Vitro and Docking Study)

**FIGURE 3:**
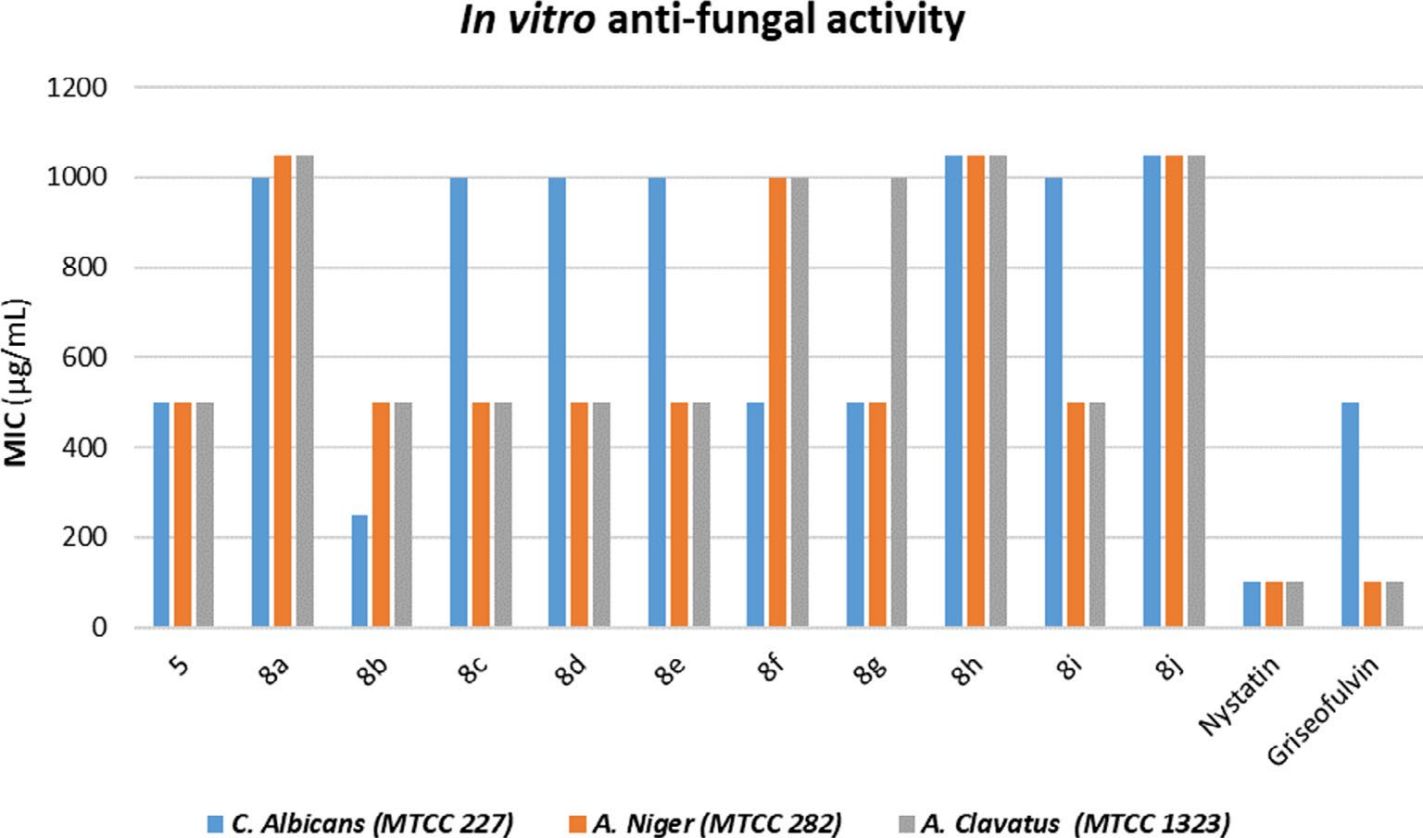
In vitro antifungal activity of compounds (**8a–j**).

The antifungal potential of the synthesized 1,2,3‐triazole *N*‐hydroxycarboxamides (**8a–j**) was evaluated against three fungal strains, namely *Candida albicans* (MTCC 227), *Aspergillus niger* (MTCC 282), and *Aspergillus clavatus* (MTCC 1323), using Nystatin and Griseofulvin as reference standard drugs (Table 4). Most of the synthesized compounds exhibited negligible antifungal activity against the tested strains, with the exception of compound **8b**, which showed mild inhibition of *C. albicans* with an MIC value of 250 μg/mL, compared to Nystatin (MIC = 100 μg/mL) and Griseofulvin (MIC = 500 μg/mL) (Figure 3). Among the *N*‐hydroxycarboxamide derivatives, compound **8b** bearing a *meta‐methyl* substituent exhibited the highest activity against all three tested strains, while compounds **8f** (*meta‐chloro*) and **8g** (*para‐methoxy*) displayed comparable levels of activity. The antifungal property of these compounds has no structural trend and relies on a delicate balance of electronic effects, lipophilicity, steric factors, and hydrogen‐bonding capability. Overall, the introduction of sulfonamide and *N*‐hydroxycarboxamide moieties into the parent 1,2,3‐triazole derivative **5** appears to deteriorate the antifungal activity, suggesting that these structural modifications are unfavorable for antifungal efficacy.

**TABLE 4:** In vitro antifungal activity of compounds (**8a–j**).

Fungal strain	*C. albicans* (MTCC 227)	*A. niger* (MTCC 282)	*A. clavatus* (MTCC 1323)
Compound			
**5**	500	500	500
**8a**	1000	> 1000	> 1000
**8b**	250	500	500
**8c**	1000	500	500
**8d**	1000	500	500
**8e**	1000	500	500
**8f**	500	1000	1000
**8 g**	500	500	1000
**8 h**	> 1000	> 1000	> 1000
**8i**	1000	500	500
**8j**	> 1000	> 1000	> 1000
Nystatin	100	100	100
Griseofulvin	500	100	100

**FIGURE 4:**
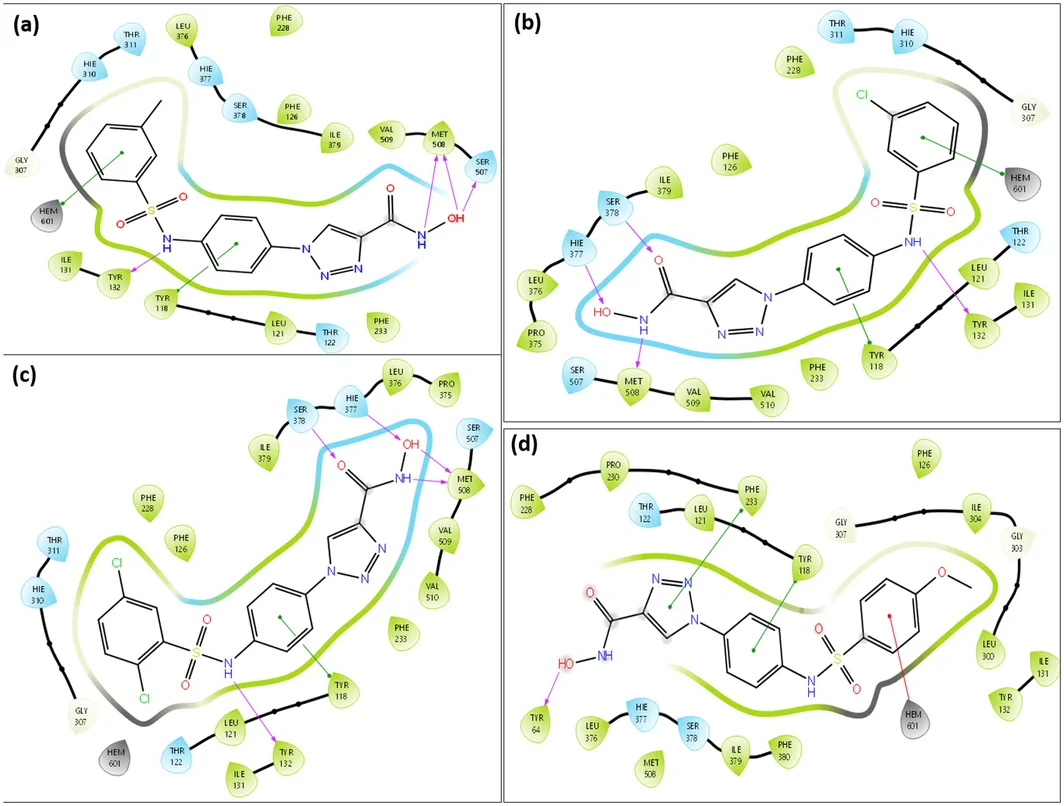
2D molecular docking interaction diagrams of the synthesized compounds (a) **8b**, (b) **8f**, (c) **8i**, and (d) **8g**, against the active site of lanosterol 14α‐demethylase (CYP51), illustrating the key ligand‐residue interactions. The corresponding 2D docking interaction diagrams of the remaining synthesized compounds and standard drugs were provided in the Figures S3 and S4.

The in vitro antifungal screening results and the molecular docking study have provided evidence to suggest that compound **8b** was the most interesting derivative among the synthesized derivatives **8a**‐**8j** (Table 5). This compound showed the best binding affinity among all derivatives, with a docking score of −6.102 kcal/mol against lanosterol 14α‐demethylase (CYP51, PDB ID = 5V5Z, Resolution = 2.90 Å) from *Candida albicans* , an important enzyme in the fungal ergosterol‐biosynthetic pathway (Keniya et al. 2018). Whereas compound **8i** showed the second‐best affinity with −6.053 kcal/mol. In energetic terms, the high binding affinity of compound **8b** was primarily supported by a lipophilic component of −5.592 kcal/mol, followed by an H‐bond contribution of −1.613 kcal/mol and an electrostatic contribution of −0.633 kcal/mol. However, there was a low penalty of 1.636 kcal/mol, which opposes the binding. In addition, docking results have indicated multiple *H*‐bonding interactions with residues MET508, SER378, and HIE377 within the binding pocket, along with hydrophobic interactions with TYR118 and HEM601 (Figure 4a–d; Figures S3 and S4). Most importantly, the binding behavior of this compound has been confirmed biologically as well, with the lowest MIC of 250 μg/mL against *C. albicans* and an MIC of 500 μg/mL against *A. niger* and *A. clavatus* . Though compound **8i** displayed a similar docking score value of −6.053 kcal/mol, the experimental activities were low, with MIC values of 1000, 500, and 500 μg/mL for *C. albicans* , *A. niger* , and *A. clavatus* , respectively. The compounds **8c**, **8d**, and **8e** displayed moderate antifungal activities with MIC values of 1000, 500, and 500 μg/mL and docking scores of −3.788, −3.245, and −1.707 kcal/mol, respectively, but the docking scores obtained by these compounds were much lower than those of **8i**. **8f** and **8g** exhibited intermediate docking scores (−4.542 and −3.797 kcal/mol, respectively) with moderate antifungal activities, while **8h** displayed the worst correlation between docking scores (+1.073 kcal/mol) and MIC values (> 1000 μg/mL against all fungal strains). In summary, all these results revealed compound **8b** as the lead compound because of its favorable docking score and highest antifungal activity among synthesized compounds **8a**‐**8j**. Nevertheless, the difference between docking affinity and MIC values for compounds such as **8i** indicates that cellular permeability, solubility, target accessibility, and other pharmacokinetic factors may also contribute to antifungal activity. Compared with the reference drugs, **8b** remains less potent than nystatin (100 μg/mL) and griseofulvin (100–500 μg/mL), but its combined computational and experimental profile supports **8b** as a promising scaffold for further structural optimization and antifungal development.

**TABLE 5:** Docking properties of ligands **8a–j** against the active site of Lanosterol 14α‐demethylase (CYP51).

Title	Docking score	Glide energy	H‐bond cont.	Lipophilic cont.	Electro‐static cont.	XP LowMW	XP rotational penalty	XP penalties	XP pose rank	Glide rotatable bonds
**8b**	−6.10	−57.52	−1.61	−5.59	−0.63	−0.26	0.36	1.64	1	7
**8i**	−6.05	−61.11	−1.40	−5.97	−0.63	−0.07	0.28	1.92	1	7
**8f**	−4.54	−63.73	−2.32	−5.68	−0.90	−0.19	0.00	4.55	1	7
**8g**	−3.80	−55.16	−0.96	−6.24	−0.02	−0.20	0.00	3.63	1	8
**8c**	−3.79	−58.95	−2.27	−5.37	−0.76	−0.24	0.00	4.85	1	7
**8a**	−3.46	−56.45	−1.62	−5.64	−0.48	−0.26	0.36	4.17	1	7
**8d**	−3.25	−60.00	−1.84	−5.20	−0.79	−0.24	0.35	4.47	1	7
**8j**	−2.94	−59.53	−1.40	−5.63	−0.83	−0.07	0.28	4.72	1	7
**8e**	−1.71	−60.44	−0.73	−5.89	−0.44	−0.19	0.32	5.34	1	7
**8h**	1.07	−64.12	−0.96	−6.18	−0.66	−0.07	0.28	8.67	1	7
Co‐crys. Lig.	−5.84	−77.72	0.00	−8.76	−0.22	0.00	0.00	4.04	1	11
Fluconazole	−5.13	−40.95	−0.70	−3.35	−0.17	−0.48	0.00	1.00	1	6
Ketoconazole	−6.13	−67.80	−0.70	−8.70	−0.24	0.00	0.00	4.01	1	8
Abafungin	−4.49	−50.62	−0.24	−7.13	−0.16	−0.24	0.00	4.00	1	5

#### 2.2.3 Antimalarial Activity

**FIGURE 5:**
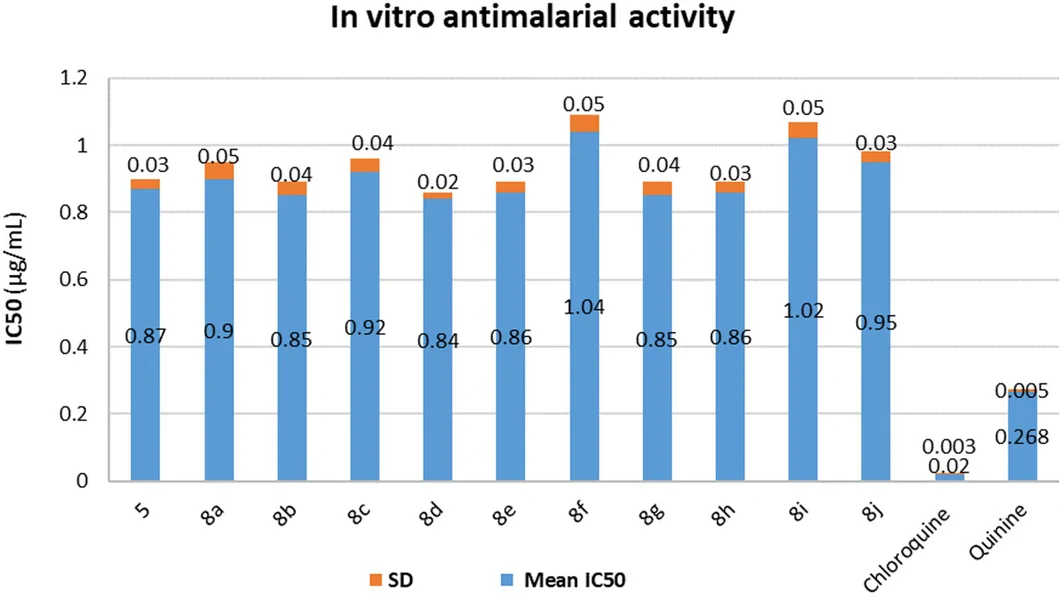
In vitro antimalarial activity of compounds (**8a–j**).

The antimalarial activity of the synthesized 1,2,3‐triazole derivatives (**8a–j**) was assessed based on their mean IC_50_ values against Plasmodium species, using chloroquine and quinine as reference drugs (Figure 5). All experiments were performed in triplicate (*n* = 3), and the results are expressed as the mean of IC50 values for parasite growth. The results demonstrated variable potencies among the tested compounds, with compound **8d** (*meta‐fluoro*) exhibiting the highest activity, with an IC_50_ value of 0.84 μg/mL. The enhanced potency of **8d** suggests that the small size and high electronegativity of fluorine may optimize electronic distribution while minimizing steric interference, thereby facilitating favorable interactions with the biological target. Compounds **8b** (*meta‐methyl*), **8e** (*para‐chloro*), **8g** (*para‐methoxy*), and **8h** (*3,4‐dichloro*) displayed comparable activities, with IC_50_ values of 0.85, 0.86, 0.85, and 0.86 μg/mL, respectively. The comparable activities of the *para*‐chloro (**8e**) and 3,4‐dichloro (**8h**) derivatives suggest that halogen substitution enhances antimalarial activity, likely by increasing lipophilicity and hydrophobic interactions. Similarly, the activity of the *meta*‐methyl (**8b**) and *para*‐methoxy (**8g**) analogues indicates that electron‐donating substituents are also favorable, possibly by optimizing membrane permeability and physicochemical properties. Although these derivatives exhibited lower potency than the standard antimalarial drugs quinine (IC_50_ = 0.268 μg/mL) and chloroquine (IC_50_ = 0.020 μg/mL), the observed structure–activity relationship provides valuable insights for the design and development of more potent 1,2,3‐triazole‐based antimalarial agents.

#### 2.2.4 Antitubercular Activity

**FIGURE 6:**
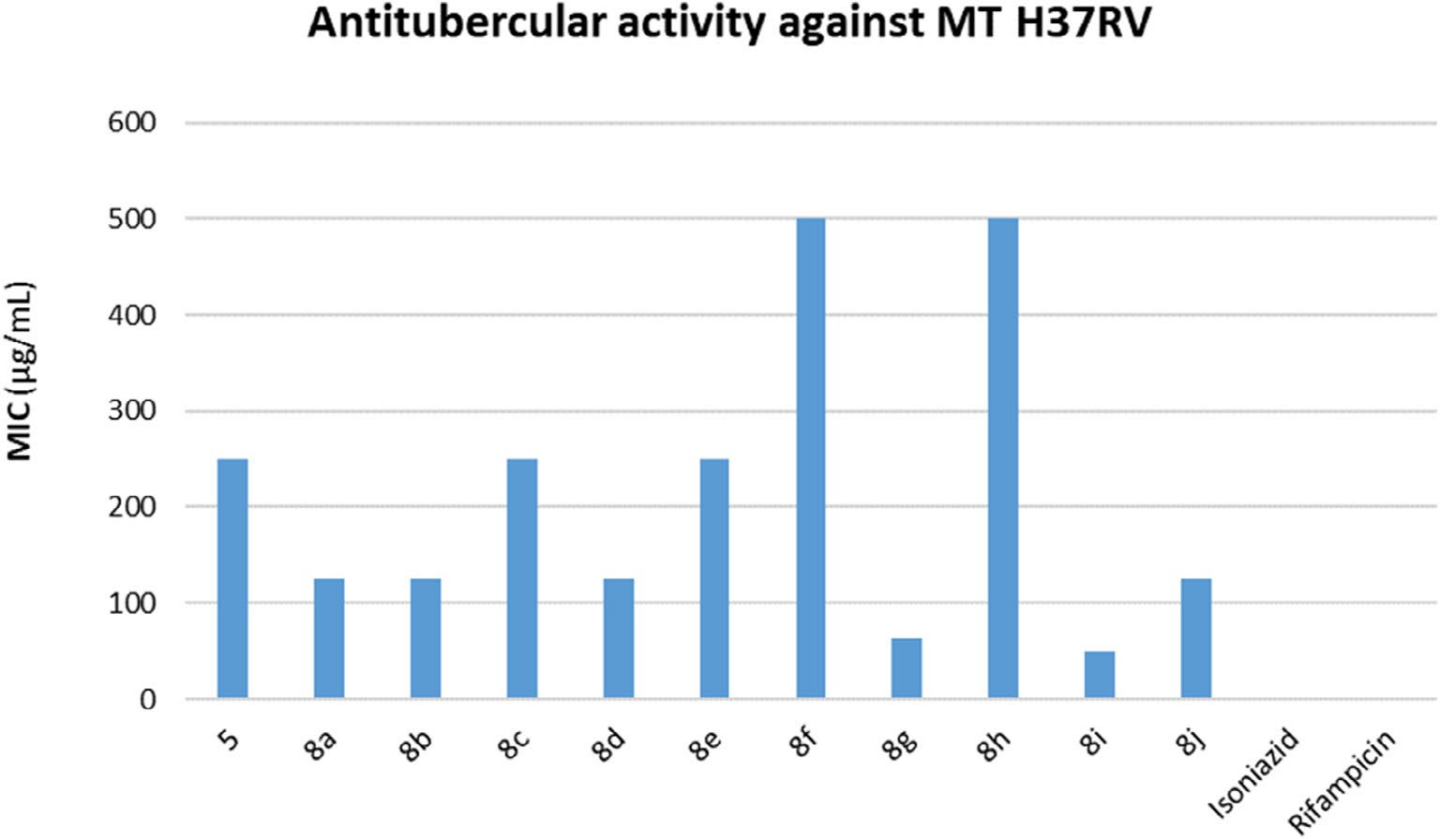
In vitro antitubercular activity of compounds (**8a–j**).

The antitubercular activity of compounds **8a–j** was assessed against *Mycobacterium tuberculosis* H37Rv using the Löwenstein‐Jensen (L.J.) medium method (Figure 6). Among the tested derivatives, **8g** (*para‐methoxy*) and **8i** (*2,5‐dichloro*) exhibited the highest inhibitory effects, with MICs of 62.5 μg/mL and 50 μg/mL, respectively. Compounds **8a**, **8b**, and **8d** also showed notable activity, each with an MIC of 125 μg/mL. None of the synthesized compounds matched the potency of the standard drugs isoniazid (MIC = 0.20 μg/mL) or rifampicin (MIC = 0.25 μg/mL). The SAR analysis indicates that both electron‐donating (CH₃ and OCH₃) and electron‐withdrawing (F and Cl) substituents can enhance antitubercular activity, depending on their position on the aromatic ring. In particular, the superior activity of the para‐methoxy and 2,5‐dichloro derivatives suggests that an optimal balance of electronic effects and lipophilicity is important for improving antimycobacterial activity. In contrast, chloro‐substitution appears to influence activity in a position‐dependent manner.

#### 2.2.5 In Silico Study of Kinase Inhibitory Activity

The synthesized compounds exhibited moderate antimicrobial, antimalarial, and antitubercular activities in the biological evaluation. In view of the well‐documented kinase inhibitory potential of nitrogen‐rich linear scaffolds, an in silico molecular docking study was undertaken to investigate the kinase‐binding characteristics of the synthesized compounds (Godesi et al. 2023; Wu et al. 2019; Davies et al. 2011). Human c‐KIT tyrosine kinase was selected as the computational target because it is a well‐characterized receptor tyrosine kinase that plays a critical role in regulating cellular proliferation, differentiation, survival, and apoptosis, and its aberrant activation has been implicated in the pathogenesis of several malignancies, including gastrointestinal stromal tumors (GISTs), melanoma, mastocytosis, and certain forms of leukemia. The crystal structure of the active kinase domain of human c‐KIT (PDB ID: 1 T46), featuring a well‐resolved ATP‐binding pocket, provides a reliable structural model for structure‐based molecular docking studies. Accordingly, the docking analysis was performed to evaluate the binding interactions and potential kinase‐binding propensity of the synthesized triazole derivatives. It should be noted that c‐KIT is not an established molecular target for the antimicrobial, antimalarial, or antitubercular activities investigated in the present study; therefore, the docking results are intended to provide insights into the kinase‐binding potential of the compounds rather than to explain the mechanism underlying their observed anti‐infective activities.

The molecular docking analysis of the synthesized 1,2,3‐triazole derivatives (**8a–j**) was carried out at the ATP‐binding site of the c‐KIT kinase domain, targeting key residues GLU671, CYS673, ASP677, and ARG796 (Pathania et al. 2021). The docking scores and key interacting amino acid residues are summarised in Table 6. The compounds exhibited a range of hydrogen‐bonding and π‐π stacking as polar interactions, as well as weak aromatic *H*‐bond interactions with the 1 T46 protein. Notably, the docking results revealed favorable binding affinities, ranging from −7.296 to −2.188 kcal/mol, accompanied by well‐oriented binding poses within the active site, compared to −6.111, −7.339, and −7.615 kcal/mol for existing drugs dasatinib, motesanib, and sunitinib, respectively. Six out of 10 ligands consistently interact with residue CYS673 as *H*‐bonding, one of the key residues of the ATP binding site. Among these compounds, **8e**, **8c**, and **8a** demonstrated highest docking scores of −7.296, −6.872, and −6.78 kcal/mol, respectively, which were better than dasatinib and comparable to motesanib and sunitinib. Analysis of the substitution pattern suggests that para‐substituted derivatives generally display more favorable binding than their corresponding meta‐substituted analogues, possibly because para substitution provides a more favorable balance of electronic effects and steric accessibility within the ATP‐binding pocket. Furthermore, halogen (F and Cl) and methyl substituents appear to promote ligand‐protein interactions through a combination of hydrophobic contacts and favorable electronic effects. In contrast, derivatives bearing electron‐withdrawing substituents at the meta position, such as **8d**, **8i**, and **8j**, exhibited comparatively lower docking scores, suggesting that this substitution pattern may be less favorable for interactions within the c‐KIT binding pocket.

**TABLE 6:** Molecular docking affinity values and interactions.

Entry	Docking score	ΔG_bind_	No of H‐ bonds	Interacting residues			
				H‐bonding (bond length in Å)	Pi‐Pi stacking (No. of interaction)	Aromatic‐H bond (No. of interaction)	Other
**8a**	**−6.78**	**−77.070**	3	CYS673 (1.94) CYS673 (2.15) ASP810 (2.50)	PHE811 (2)	GLU640 (1) ILE808 (1)	—
**8b**	−6.009	−69.428	2	CYS673 (1.99) ASP810 (2.39)	PHE811 (1)	GLU640 (1)	—
**8c**	**−6.872**	**−70.367**	3	CYS673 (1.96) CYS673 (2.11) ASP810 (2.51)	PHE811 (1)	GLU640 (1) ILE808 (1)	—
**8d**	−4.527	−67.314	1	LEU595 (2.00)	PHE811 (1)	VAL668 (2) ALA621 (1)	—
**8e**	**−7.296**	**−73.695**	3	CYS673 (1.94) CYS673 (2.15) ASP810 (2.50)	PHE811 (2)	GLU640 (1) ILE808 (1)	—
**8f**	−5.093	−75.794	3	LEU595 (2.26) LYS623 (2.59) ASP810 (2.72)	PHE811 (1)	GLU640 (1) ILE808 (1)	—
**8g**	−6.709	−77.901	3	CYS673 (1.92) CYS673 (2.23) ASP810 (2.63)	PHE811 (2)		—
**8h**	−6.694	−86.708	2	CYS673 (2.11) ASP810 (2.54)	PHE811 (2)	ILE808 (1)	—
**8i**	−4.257	−55.135	1	LYS623 (2.11)	—	GLU640 (1) HIE790 (1) ASP810 (1)	—
**8j**	−2.188	−70.846	2	LYS593 (1.82) CYS674 (2.01)	—	—	LYS623 (1)^#^ VAL668 (1)* ASP810 (1)*
Dasatinib	−6.111	−100.857	2	ASN680 (2.36) LYS623 (2.62)	PHE811 (1)	—	LYS623 (1)^#^ ASP810 (1)*
Motesanib	−7.339	−85.730	3	LYS623 (2.49) ASP810 (2.01) ILE808 (2.69)	—	GLU640 (1) GLU671 (1) ILE808 (1)	LYS623 (1)^#^
Sunitinib	−7.615	−83.730	1	CYS674 (1.97)	—	—	—

^#^ Pi‐cation interaction.

* Halogen bonding.

**FIGURE 7:**
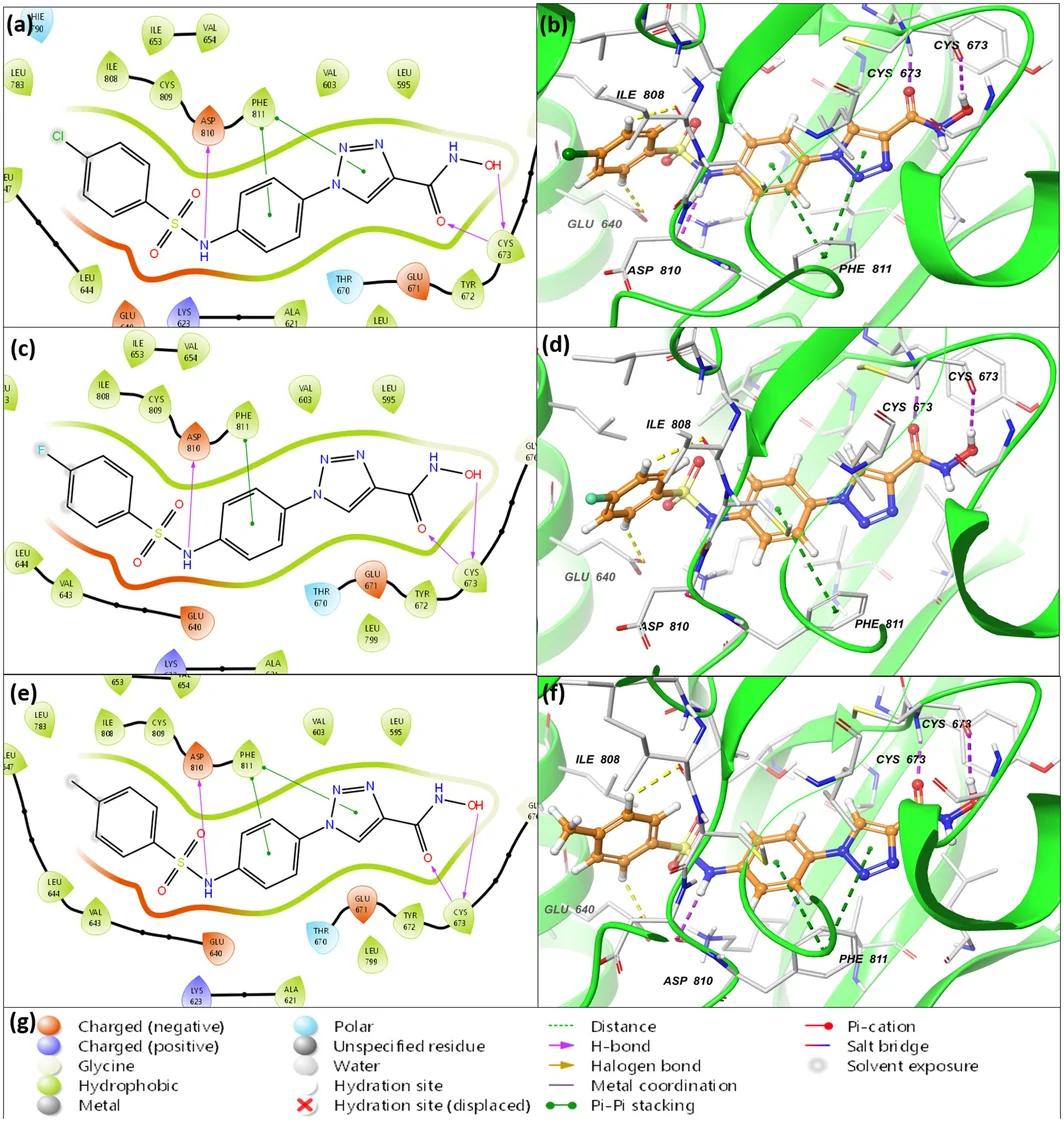
(a, c, e) Molecular Docking 2D interaction output and (b, d, f) 3D interaction output of compound **8e**, **8c**, and **8a**, respectively, against Human c‐KIT tyrosine kinase (PDB: 1 T46). (g) colour representation for different type of interactions.

All these three ligands comprise two H‐bonds with residue CYS673 and one H‐bond with ASP810, highlighting their relevance in stabilizing ligand–protein complexes (Figure 7). Along with these, π‐π stacking interactions with PHE811 and aromatic H‐bond interactions with GLU640 and ILE808 also strengthen the protein‐ligand complex. To elucidate these interactions, 2D interaction profiles and 3D binding conformations of compounds **8e**, **8c**, and **8a** were illustrated in Figure 7a–f. The interaction fingerprint pattern of the synthesized ligands closely resembled that of the reference inhibitors Dasatinib, Motesanib, and Sunitinib (Figure S5). GLU671 and CYS673 exhibited the highest interaction frequency with most ligands, except **8d**, **8f**, and **8i**, indicating their crucial role as anchoring residues in the ATP‐binding pocket. Notably, interactions with the hinge‐region residue CYS673 suggest effective binding of the triazole scaffold within the kinase active site, similar to known c‐KIT inhibitors, thereby contributing to enhanced binding affinity and inhibitory potential. Additionally, interactions with ASP677 and ARG796 further strengthened ligand accommodation within the kinase pocket.

To further validate the docking results and estimate the binding free energy (ΔG_bind_) of the protein–ligand complexes, MM/GBSA calculations were performed for all docked conformations. All evaluated ligands demonstrated energetically favorable ligand‐protein complexes, with ΔG_bind_ values ranging from −86.71 to −55.135 kcal/mol (Table 6). Among these, the shortlisted ligands **8e**, **8c**, and **8a** have ΔG_bind_ values of −73.69, −70.37, and −77.07 kcal/mol, respectively. Detailed analysis of ΔG_bind_ revealed (Table S1) that binding energetics were predominantly governed by favorable van der Waals interactions (ΔG_vdW_) and lipophilic contributions (ΔG_Lipo_), highlighting the importance of hydrophobic interactions in stabilizing the ligand within the kinase binding pocket. Hydrogen bonding contributions (ΔG_Hbond_) were relatively small for all compounds, suggesting that hydrophobic and dispersion interactions play a more dominant role in ligand recognition by c‐KIT kinase. Additionally, the solvation energy (ΔG_Solv‐GB_) also supports stable complex formation.

**FIGURE 8:**
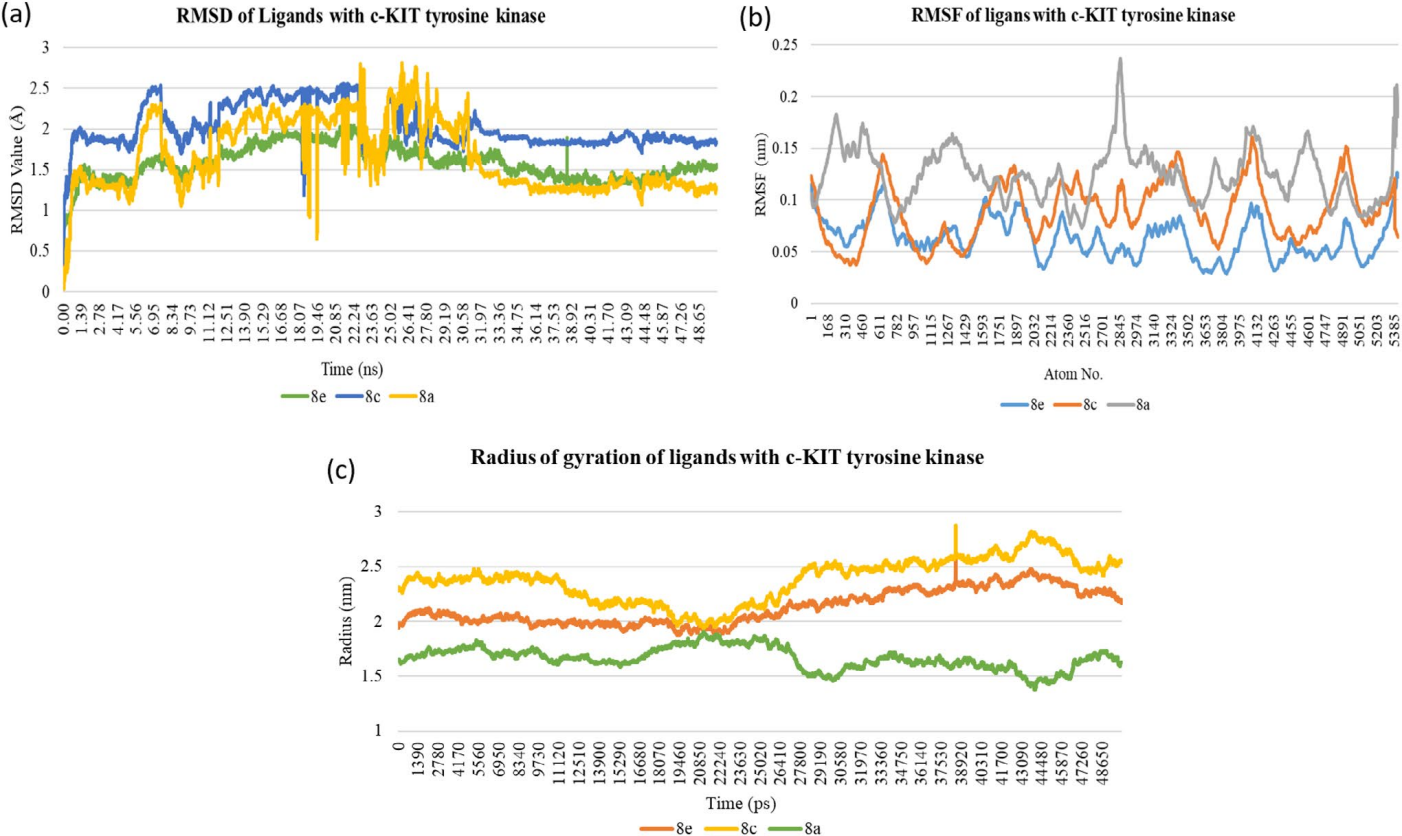
RMSD (a), RMSF (b), and the radius of gyration (c) plot for all three ligands **8e**, **8c**, and **8a** with Human c‐KIT tyrosine kinase, respectively.

The RMSD, RMSF, and radius of gyration were analyzed over the simulation time to assess the structural stability and flexibility of ligands **8e**, **8c**, and **8a** at the ATP‐binding site of the c‐KIT kinase under aqueous conditions (Figure 8). The RMSD trajectories of c‐KIT kinase with **8e** and **8c** complexes showed stabilization around a plateau near 1.4 nm, indicating favorable structural convergence (Figure 8a). In contrast, the c‐KIT kinase with the **8a** complex exhibited slightly elevated RMSD fluctuation but remained consistently below 2.8 nm, suggesting sustained conformational stability throughout the simulation. The RMSF profiles revealed average residue fluctuations between 0.03 and 0.24 nm for all three complexes, indicating limited local flexibility and overall structural stability (Figure 8b). Importantly, a lower RMSF value indicates less flexibility of amino acid residues, suggesting that ligand binding conferred localized stabilization. The radius of gyration analysis further supported the structural stability of the protein‐ligand complexes, with radius of gyration values fluctuating within a narrow range of 1.4 to 2.8 nm, and did not undergo significant unfolding or expansion (Figure 8c). The absence of sharp deviations in the radius of gyration profile indicates that no significant unfolding or global conformational changes occurred during the simulation.

Although compounds **8e**, **8c**, and **8a** exhibited the most favorable docking scores against human c‐KIT kinase, the highest experimental antimicrobial, antimalarial, and antitubercular activities were observed for compounds **8f**, **8g**, **8i**, and **8d**. This apparent lack of correlation is not unexpected, as the molecular docking study was performed using human c‐KIT kinase to evaluate the binding characteristics of the synthesized compounds within a well‐characterized kinase active site, whereas the biological assays were conducted against microbial and parasitic organisms for which c‐KIT is not an established molecular target.

### 2.3 ADME and Toxicity Study

The ADME properties of the synthesized triazole‐sulfonamide derivatives were evaluated using QikProp descriptors to assess their drug‐likeness and pharmacokinetic suitability (Table 7). The molecular weights of the synthesized compounds ranged from 373.39 to 428.25 Da, satisfying Lipinski's recommended limit (< 500 Da), which suggests favorable oral bioavailability. All compounds possessed 3 hydrogen‐bond donor groups and approximately 10–11 hydrogen‐bond acceptors, indicating balanced hydrogen‐bonding capability necessary for effective receptor binding and membrane permeability.

**TABLE 7:** ADME properties of compounds **8a–j**.

Ligand	Stars	Mol MW	SASA	Donor HB	Accpt HB	QP polrz	QP log_Po/w_	QP log_S_	QP log_HERG_	QP P_Caco_	QP log_BB_	QP P_MDCK_	QP log_Khsa_	%HOA
**8a**	1.00	373.4	650.9	3.0	10.7	38.05	0.30	−3.78	−6.27	37.6	−2.41	14.46	−0.55	56.86
**8b**	0.00	373.4	656.9	3.0	10.7	38.28	0.28	−3.87	−6.35	33.3	−2.50	12.70	−0.54	55.87
**8c**	1.00	377.3	634.5	3.0	10.7	36.74	0.25	−3.75	−6.33	33.2	−2.34	22.89	−0.63	55.63
**8d**	1.00	377.3	632.5	3.0	10.7	36.66	0.25	−3.72	−6.30	34.4	−2.31	23.78	−0.63	55.91
**8e**	1.00	393.8	650.1	3.0	10.7	37.80	0.48	−4.08	−6.37	33.0	−2.32	30.97	−0.57	56.95
**8f**	1.00	393.8	644.5	3.0	10.7	37.57	0.49	−4.00	−6.30	36.8	−2.24	34.84	−0.58	57.85
**8 g**	0.00	389.4	662.7	3.0	11.4	38.29	0.10	−3.59	−6.35	33.1	−2.56	12.61	−0.67	54.73
**8 h**	1.00	428.2	667.8	3.0	10.7	38.94	0.89	−4.60	−6.25	34.4	−2.18	67.40	−0.50	59.64
**8i**	2.00	428.2	660.5	3.0	10.7	38.71	0.86	−4.45	−6.18	37.3	−2.13	66.24	−0.50	60.08
**8j**	1.00	428.2	673.5	3.0	10.7	39.07	0.94	−4.74	−6.27	33.2	−2.20	77.03	−0.49	59.70

The solvent accessible surface area (SASA) values ranged between 632.49 and 673.48 Å^2^, reflecting moderate molecular surface exposure and suitable interaction potential with the biological environment. Similarly, the predicted polarizability (QP_polrz_) values suggested acceptable molecular flexibility and electronic distribution for receptor interaction. Lipophilicity, represented by QPlog_Po/w_, varied from 0.10 to 0.94, indicating moderate hydrophobic character. Such balanced lipophilicity may facilitate membrane permeation while minimizing excessive nonspecific binding. The aqueous solubility values (QPlog_S_) ranged from −3.59 to −4.74, suggesting acceptable solubility for most compounds, although compounds **8h**‐**8j** with increased halogen substitution showed slightly lower solubility.

The predicted intestinal permeability values (QPP_Caco_) ranged from 32.97 to 37.57 nm/s, suggesting moderate absorption potential across intestinal epithelial membranes. Likewise, MDCK cell permeability values (QPP_MDCK_) demonstrated moderate membrane permeability, particularly for compounds 8 h‐8j, which showed comparatively higher permeability values. The blood–brain barrier penetration values (QPlog_BB_) remained within acceptable limits (−2.56 to −2.13), indicating limited central nervous system penetration, which may reduce the possibility of CNS‐related side effects. The predicted human oral absorption (%HOA) values ranged from 54.73% to 60.08%, indicating moderate oral absorption characteristics for the synthesized molecules. The synthesized compounds exhibited generally acceptable drug‐like characteristics, satisfying Lipinski's rule of five and several key ADME criteria. However, the predicted QPlog_HERG_ values (−6.18 to −6.37) suggest a potential risk of hERG channel inhibition, while the predicted human oral absorption (55%–60%) indicates moderate oral bioavailability. Therefore, although the compounds demonstrate promising in silico pharmacokinetic profiles, these predictions should be interpreted with caution and require further experimental validation.

## 3 Conclusion

The research conducted in this study has led to the successful synthesis of a new collection of *N*‐hydroxy‐1‐(4‐(aryl sulfonamido)phenyl)‐1*H*‐1,2,3‐triazole‐4‐carboxamide derivatives (**8a–j**) through a multistep strategy that incorporated the three main pharmacophoric motifs, namely aryl sulfonamide, *N*‐hydroxycarboxamide, and 1,2,3‐triazole. The biological evaluation brought to light the multifunctional potential of these derivatives. A number of compounds displayed moderate antibacterial potency, notably **8f**, **8g**, and **8i**, which demonstrated activity against both Gram‐positive and Gram‐negative bacterial strains. While the majority of the compounds possessed only slight antifungal activity, the assays for antibacterial, antimalarial, and antitubercular activity brought to the fore the promising leading structures. Among them, **8d** exhibited the strongest antimalarial potency (IC_50_ = 0.84 μg/mL), while **8g** (MICs = 62.5 μg/mL) and **8i** (MICs = 50 μg/mL) were indicated as the most potent antitubercular agents. These results highlight the critical role of substituent effects, in particular those of the fluoro, methoxy, and methyl groups, on the biological response of the sulfonamide‐modified triazole framework. The molecular docking study against DNA gyrase (PDB ID: 7FVT) and lanosterol 14α‐demethylase (PDB ID: 5V5Z) as relevant antibacterial and antifungal targets, respectively, showed a similar trend of activity.

Moreover, in silico studies have provided additional support for their biological activity against human c‐KIT tyrosine kinase. The binding affinities and ΔG_bind_ of molecular docking and MM/GBSA studies of compounds **8e**, **8c**, and **8a** with the c‐KIT (1 T46) kinase were quite good, with each of them making strong interactions with the key residues in the active site, GLU671, CYS673, and ASP810, and comparable to those of the standard kinase inhibitors. The ADME analysis indicated that the synthesized compounds exhibit drug‐like properties and acceptable pharmacokinetic profiles, with possible moderate cardiotoxicity and moderate oral bioavailability.

In summary, the integration of sulfonamide, triazole, and *N‐*hydroxycarboxamide functionalities through rational design has resulted in the formation of different molecules with considerable biological potential, establishing the basis for further optimization. The hybridization approach utilized in this research creates a flexible platform for designing multifunctional agents, and ongoing optimization may result in clinically significant candidates with wide‐ranging biological activities.

## 4 Experimental Section

### 4.1 Material and Method

Commercially available reagents and solvents obtained from TCI Chemicals, Sigma‐Aldrich, and Alfa Aesar were used without further purification. All reactions were performed in dry glassware, and completion of the reaction was monitored by analytical thin‐layer chromatography (TLC). Extraction and chromatographic separations were carried out using reagent‐grade solvents, and yields refer to chromatographically purified products. Infrared (IR) spectra were recorded using a Bruker Alpha II Platinum ATR spectrometer, with stretching frequencies reported in wavenumbers (cm^−1^). Nuclear magnetic resonance (NMR) spectra were acquired on Bruker 400 MHz (^1^H) and 100 MHz (^13^C) spectrometers. DMSO‐d₆ (Sigma‐Aldrich) was used as the NMR solvent, and chemical shifts are reported in parts per million (ppm) relative to the residual DMSO‐d₆ signal for ^1^H (δ 2.50 ppm) and ^13^C (δ 39.52 ppm). Signal multiplicities are indicated as: s = singlet, d = doublet, dd = doublet of doublets, t = triplet, m = multiplet, and bs = broad singlet. Coupling constants (J) are reported in hertz (Hz). High‐resolution mass spectra (HRMS) were obtained using an Agilent G6530AA quadrupole time‐of‐flight (LC‐HRMS‐Q‐TOF) instrument.

The detailed synthetic procedures for compounds **2–7** are provided in the Supporting Information S1. The synthetic procedure and characterization details for the final compounds **8a–j** were presented below.

### 4.2 General Procedure for Synthesis of Final Compounds 8a‐j

To a solution of compounds (**7a–j**, 0.3 mmole, 1.0 eq.) in ethanol (10.0 mL) was added hydroxyl amine hydrochloride (1.5 eq), followed by powdered potassium hydroxide (3.0 eq). The resultant reaction mixture was heated to 75°C–80°C and continued stirring for 6 h. The completion of the reaction was monitored by TLC. After completion of the reaction, the reaction mass was evaporated under reduced pressure till dry. The residue was taken in water (10 mL) and acidified to pH 3–4 with conc. HCl; a solid precipitated out. Filter the resultant solid and dry under reduced pressure. The resulting crude material was purified by column chromatography using silica gel and 5%–10% methanol in dichloromethane as the eluent, yielding the desired solid product (**8a–j**) with a yield range of 72%–85%.

### 4.3 8a. *N*‐Hydroxy‐1‐(4‐((4‐Methylphenyl)sulfonamido)phenyl)‐1*H* ‐1,2,3‐Triazole‐4‐Carboxamide

Yield: 81% (91 mg); Off white solid, mp 168°C–170°C; IR (KBr) 3631, 3156, 2851, 1732, 1641, 1335, 1230, 1181 cm^−1^; ^1^H‐NMR (400 MHz, DMSO‐d_6_) *δ* 2.33 (s, 3H, CH_3_), 7.28 (d, *J* = 8.8 Hz, 2H, ArH), 7.36 (d, *J* = 8.1 Hz, 2H, ArH), 7.70 (d, *J* = 8.3 Hz, 2H, ArH), 7.80 (d, *J* = 9.0 Hz, 2H, ArH), 9.11–9.13 (2 s, 2H, ArH and NH), 10.62 (s, 1H, NH), 11.40 (s, 1H, OH); ^13^C{^1^H} NMR (100 MHz, DMSO‐d_6_): *δ* 20.96, 120.25, 121.55, 124.31, 126.73, 129.85, 132.04, 136.43, 138.58, 142.14, 143.58, 157.26; HRMS (ESI): calculated for C_16_H_16_N_5_O_4_S, 374.0918 (MH^+^); found: *m/z*, 374.0910.

### 4.4 8b. *N*‐Hydroxy‐1‐(4‐((3‐Methylphenyl)sulfonamido)phenyl)‐1*H* ‐1,2,3‐Triazole‐4‐Carboxamide

Yield: 75% (84 mg); Off white solid, mp 153°C–155°C; IR (KBr) 3161, 2827, 1645, 1516, 1342, 1227, 1156 cm^−1^; ^1^H‐NMR (400 MHz, DMSO‐d_6_) *δ* 2.37 (s, 3H, CH_3_), 7.31 (dd, *J* = 7.0, 1.9 Hz, 2H, ArH), 7.46–7.49 (m, 2H, ArH), 7.62–7.65 (m, 2H, ArH), 7.65 (s, 1H, ArH), 7.83 (dd, *J* = 9.0, 1.9 Hz, 2H, ArH), 9.13–9.15 (2 s, 2H, ArH and NH), 10.66 (bs, 1H, NH), 11.39 (s, 1H, OH); ^13^C{^1^H} NMR (100 MHz, DMSO‐d_6_): *δ* 20.83, 120.29, 121.57, 123.85, 124.33, 126.85, 129.27, 132.11, 133.82, 138.47, 139.18, 139.27, 142.15, 157.28; HRMS (ESI): calculated for C_16_H_16_N_5_O_4_S, 374.0918 (MH^+^); found: *m/z*, 374.0910.

### 4.5 8c. 1‐(4‐((4‐Fluorophenyl)sulfonamido)phenyl)‐*N*‐Hydroxy‐1*H* ‐1,2,3‐Triazole‐4‐Carboxamide

Yield: 79% (90 mg); Off white solid, mp 181°C–183°C; IR (KBr) 3138, 2874, 1728, 1644, 1578, 1339, 1230, 1164 cm^−1^; ^1^H‐NMR (400 MHz, DMSO‐d_6_ + D_2_O) 7.27 (d, *J* = 8.7 Hz, 2H, ArH), 7.35 (t, *J* = 8.3 Hz, 2H, ArH), 7.76 (d, *J* = 7.2 Hz, 2H, ArH), 7.85 (dd, *J* = 8.4, 5.1 Hz, 2H, ArH), 8.97 (d, *J* = 4.0 Hz, 1H, ArH); NH and OH peak at 9.34 and 11.46 ppm were disappeared due to deuterium exchange; D_2_O was added to DMSO to improve the compound's solubility and ensure a homogeneous solution; ^13^C{^1^H} NMR (100 MHz, DMSO‐d_6_ + D_2_O): *δ* 116.71 (d, *J* = 22 Hz), 121.66 (d, *J* = 89 Hz), 124.38, 129.85 (d, *J* = 9 Hz), 132.34, 135.74, 138.41, 142.20, 157.35, 164.48(d, *J* = 250 Hz); HRMS (ESI): calculated for C_15_H_13_FN_5_O_4_S, 378.0667 (MH^+^); found: *m/z*, 378.0669.

### 4.6 8d. 1‐(4‐((3‐Fluorophenyl)sulfonamido)phenyl)‐*N*‐Hydroxy‐1*H* ‐1,2,3‐Triazole‐4‐Carboxamide

Yield: 76% (86 mg); Off white solid, mp 170°C–172°C; IR (KBr) 3169, 2879, 1643, 1519, 1348, 1225, 1150 cm^−1^; ^1^H‐NMR (400 MHz, DMSO‐d_6_) *δ* 7.30 (d, *J* = 8.9 Hz, 2H, ArH), 7.50–7.54 (m, 1H, c), 7.60–7.66 (m, 3H, ArH), 7.84 (d, *J* = 8.9 Hz, 2H, ArH), 9.13–9.14 (2 s, 2H, ArH and NH), 10.79 (bs, 1H, NH), 11.40 (s, 1H, OH); ^13^C{^1^H} NMR (100 MHz, DMSO‐d_6_): *δ* 113.76 (d, *J* = 24 Hz), 120.36, 120.57, 121.25 (d, *J* = 81 Hz), 123.02, 124.38, 131.91, 132.49, 138.02, 141.28 (d, *J* = 6.3 Hz), 142.19, 157.28, 161.70 (d, *J* = 249 Hz); HRMS (ESI): calculated for C_15_H_13_FN_5_O_4_S, 378.0667 (MH^+^); found: *m/z*, 378.0668.

### 4.7 8e. 1‐(4‐((4‐Chlorophenyl)sulfonamido)phenyl)‐*N*‐Hydroxy‐1*H* ‐1,2,3‐Triazole‐4‐Carboxamide

Yield: 83% (98 mg); Off white solid, mp 191°C–193°C; IR (KBr) 3579, 3347, 3136, 2852, 1646, 1332, 1223, 1158 cm^−1^; ^1^H‐NMR (400 MHz, DMSO‐d_6_) *δ* 7.28 (d, *J* = 7.9 Hz, 2H, ArH), 7.65 (d, *J* = 8.2 Hz, 2H, ArH), 7.82 (m, 4H, ArH), 9.12 (s, 2H, ArH and NH), 10.76 (bs, 1H, NH), 11.40 (s, 1H, OH); ^13^C{^1^H} NMR (100 MHz, DMSO‐d_6_): *δ* 120.82, 121.61, 124.30, 128.64, 129.62, 132.34, 132.41, 138.09, 138.16, 142.19, 157.26; HRMS (ESI): calculated for C_15_H_13_ClN_5_O_4_S, 394.0371 (MH^+^); found: *m/z*, 394.0368.

### 4.8 8f. 1‐(4‐((3‐Chlorophenyl)sulfonamido)phenyl)‐*N*‐Hydroxy‐1*H* ‐1,2,3‐Triazole‐4‐Carboxamide

Yield: 78% (92 mg); Off white solid, mp 180°C–182°C; IR (KBr) 3164, 3133, 2851, 1643, 1349, 1229, 1162 cm^−1^; ^1^H‐NMR (400 MHz, DMSO‐d_6_) *δ* 7.30 (d, *J* = 8.3 Hz, 2H, ArH), 7.61 (t, *J* = 7.9 Hz, 1H, ArH), 7.74 (t, *J* = 7.8 Hz, 2H, ArH), 7.82–7.85 (m, 3H, ArH), 9.14 (s, 2H, ArH and NH), 10.79 (bs, 1H, NH), 11.40 (s, 1H, OH); ^13^C{^1^H} NMR (100 MHz, DMSO‐d_6_): *δ* 120.88, 121.65, 124.37, 125.43, 126.22, 131.56, 132.54, 133.23, 134.00, 137.89, 141.07, 142.19, 157.24; HRMS (ESI): calculated for C_15_H_13_ClN_5_O_4_S, 394.0371 (MH^+^); found: *m/z*, 394.0368.

### 4.9 8g. *N*‐Hydroxy‐1‐(4‐((3‐Methoxyphenyl)sulfonamido)phenyl)‐1*H* ‐1,2,3‐Triazole‐4‐Carboxamide

Yield: 85% (100 mg); Off white solid, mp 173°C–175°C; IR (KBr) 3643, 3153, 2847, 1645, 1336, 1231, 1154 cm^−1^; ^1^H‐NMR (400 MHz, DMSO‐d_6_) *δ* 3.79 (s, 3H, OCH_3_), 7.07 (d, *J* = 8.9 Hz, 2H, ArH), 7.27 (d, *J* = 8.9 Hz, 2H, ArH), 7.74 (d, *J* = 8.9 Hz, 2H, ArH), 7.80 (d, *J* = 8.9 Hz, 2H, ArH), 9.12 (2 s, 2H, ArH and NH), 10.54 (bs, 1H, NH), 11.39 (bs, 1H, OH); ^13^C{^1^H} NMR (100 MHz, DMSO‐d_6_): *δ* 60.04, 116.10, 116.88, 122.80, 124.84, 127.94, 130.59, 132.84, 133.64, 135.71, 145.60, 158.97; HRMS (ESI): calculated for C_16_H_16_N_5_O_5_S, 390.0867 (MH^+^); found: *m/z*, 390.0867.

### 4.10 8h. 1‐(4‐((3,4‐Dichlorophenyl)sulfonamido)phenyl)‐*N*‐Hydroxy‐1*H* ‐1,2,3‐Triazole‐4‐Carboxamide

Yield: 76% (97 mg); Off white solid, mp 214°C–216°C; IR (KBr) 3163, 2873, 1646, 1517, 1356, 1230, 1168 cm^−1^; ^1^H‐NMR (400 MHz, DMSO‐d_6_ + D_2_O) δ 7.28 (d, *J* = 8.9 Hz, 2H, ArH), 7.71 (d, *J* = 1.7 Hz, 2H, ArH), 7.76–7.78 (m, 3H, ArH), 8.94 (s, 1H, ArH); NH and OH peaks at 9.18, 9.40 and 11.46 ppm disappeared due to deuterium exchange; D_2_O was added to DMSO to improve the compound's solubility and ensure a homogeneous solution; ^3^C{^1^H} NMR (100 MHz, DMSO‐d_6_ + D_2_O): *δ* 121.18, 121.68, 124.36, 126.80, 128.43, 131.90, 132.36, 132.70, 136.36, 137.72, 139.51, 142.20, 157.28; HRMS (ESI): calculated for C_15_H_11_Cl_2_N_5_O_4_S, 426.9909 (MH^+^); found: *m/z* 426.9901.

### 4.11 8i. 1‐(4‐((2,5‐Dichlorophenyl)sulfonamido)phenyl)‐*N*‐Hydroxy‐1*H* ‐1,2,3‐Triazole‐4‐Carboxamide

Yield: 72% (92 mg); Off white solid, mp 190°C–192°C; IR (KBr) 3281, 3141, 2883, 1660, 1330, 1249, 1161,1037 cm^−1^; ^1^H‐NMR (400 MHz, DMSO‐d_6_ + D_2_O) δ 7.30 (d, J = 9.0 Hz, 2H, ArH), 7.62–7.68 (m, 2H, ArH), 7.77 (d, J = 9.0 Hz, 2H, ArH), 8.02 (d, J = 2.3 Hz, 1H), 8.96 (s, 1H, ArH); NH and OH peak at 9.18, 9.35 and 11.45 ppm were disappeared due to deuterium exchange; D_2_O was added to DMSO to improve the compound's solubility and ensure a homogeneous solution; ^13^C{^1^H} NMR (100 MHz, DMSO‐d_6_ + D_2_O): *δ* 120.00, 121.75, 124.38, 124.44, 129.56, 130.84, 132.34, 133.78, 134.73, 137.29, 137.78, 142.15, 157.26; HRMS (ESI): calculated for C_15_H_11_Cl_2_N_5_O_4_S, 426.9909 (MH^+^); found: *m/z*, 426.9901.

### 4.12 8j. 1‐(4‐((3,5‐Dichlorophenyl)sulfonamido)phenyl)‐*N*‐Hydroxy‐1*H* ‐1,2,3‐Triazole‐4‐Carboxamide

Yield: 75% (96 mg); Off white solid, mp 205°C–207°C; IR (KBr) 3168, 3073, 2851, 1644, 1353, 1226, 1167 cm^−1^; ^1^H‐NMR (400 MHz, DMSO‐d_6_) *δ* 7.32 (d, *J* = 8.9 Hz, 2H, ArH), 7.78 (d, *J* = 1.8 Hz, 2H, ArH), 7.87 (d, *J* = 8.9 Hz, 2H, ArH), 7.97 (t, *J* = 1.8 Hz, 1H, ArH), 9.15 (s, 2H, ArH and NH), 10.86 (bs, 1H, NH), 11.40 (s, 1H, OH); ^13^C{^1^H} NMR (100 MHz, DMSO‐d_6_): *δ* 121.25, 121.74, 124.41, 125.25, 132.81, 132.96, 135.28, 137.54, 142.21, 142.31, 157.28; HRMS (ESI): calculated for C_15_H_11_Cl_2_N_5_O_4_S, 426.9909 (MH^+^); found: *m/z*, 426.9902.

## Author Contributions

**Dineshkumar Pagar:** investigation, methodology, writing – original draft, validation, data curation, formal analysis, conceptualization, software. **Ranjay Shaw:** writing – review and editing, software, data curation. **Hemant Suryavanshi:** data curation, formal analysis. **Dhanji Rajani:** validation, investigation. **Sudhakar Patil:** conceptualization, funding acquisition, supervision, writing – review and editing.

## Funding

The work was supported by the University Grant Commission, New Delhi (Grant numbers F.No. 39‐722/2010(SR)).

## Conflicts of Interest

The authors declare no conflicts of interest.

## Supporting information


**Figure S1:** Two‐dimensional molecular docking interaction diagrams of the synthesized compounds (a) **8b**, (b) **8c**, (c) **8d**, (d) **8e**, (e) **8h**, and (f) **8i** within the active site of DNA gyrase, illustrating the key ligand–residue interactions involved in their predicted binding.
**Figure S2:** 2D molecular docking interaction diagrams of the synthesized compounds (a) co‐crystallized ligand, (b) cephalexin, (c) amoxicillin, and (d) cefixime within the active site of DNA gyrase. (e) Color‐coded legend describing the residue types and interaction categories represented in the docking diagrams.
**Figure S3:** 2D molecular docking interaction diagrams of the synthesized compounds (a) **8a**, (b) **8c**, (c) **8d**, (d) **8e**, (e) **8h**, and (f) **8i** within the active site of *lanosterol 14α‐demethylase (CYP51)*, illustrating the key ligand‐residue interactions involved in their predicted binding.
**Figure S4:** 2D molecular docking interaction diagrams of the synthesized compounds (a) co‐crystallised ligand, (b) Fluconazole, (c) Ketoconazole, and (e) Abafungin within the active site of *lanosterol 14α‐demethylase (CYP51)*. (d) Color‐coded legend describing the residue types and interaction categories represented in the docking diagrams.
**Figure S5:** Interaction fingerprint of triazole ligands with c‐KIT tyrosine kinase.
**Table S1:** mmGBSA‐Study of ligands with c‐KIT tyrosine kinase.

## Data Availability

The data that support the findings of this study are available in the Supporting Information S1 of this article.
