# Supplementary material for: Design and Synthesis of Novel 1,2,3‐Triazole Conjugates Featuring Hydroxamic Acid and Sulfonamide Moieties: Exploring Dual Pharmacophores for Enhanced Biological Activity
Source: Chem Biol Drug Des. 2026 Sep 17;108(3):e70400. doi: 10.1111/cbdd.70400 (PMC13583908; doi:10.1111/cbdd.70400)
Supplement: Supplementary file 1 [file CBDD-108-e70400-s001.docx]

**Design and Synthesis of Novel 1,2,3-Triazole Conjugates Featuring Hydroxamic Acid and Sulfonamide Moieties: Exploring Dual Pharmacophores for Enhanced Biological Activity**

Dineshkumar Pagar,^a,*^ Ranjay Shaw,^b^ Hemant Suryavanshi,^c^ Dhanji Rajani,^d^ Sudhakar Patil^a,*^

^a^ **Organic Chemistry Research Laboratory, Maharashtra Udaygiri Mahavidyalay, Swami Ramanand Teerth Marathwada University**, Nanded, India.

^b^ Department of Chemistry, GLA University, Mathura, U.P., 281406.

^c^ Zeal College of Engineering and Research, Narhe, Pune, 411041.

^d^ Microcare Laboratory, Surat, 395001, India.

* Corresponding Author:

Dineshkumar Pagar - [dk.pagar@gmail.com](mailto:dk.pagar@gmail.com); Sudhakar Patil - [patil.sudhakar261@gmail.com](mailto:patil.sudhakar261@gmail.com)

| **Sr. No.** | **Title** | **Page No.** |
| --- | --- | --- |
| **1.** | **Synthesis of precursors 2 to 5** | **2** |
| **2.** | **Synthesis and characterisation data of precursor 7a-j** | **3-5** |
| **3.** | **Biological Activity** | **5-9** |
| **4.** | **References** | **9** |
| **5.** | **NMR Spectra** | **10-22** |

**Synthesis of intermediate 1-azido-4-nitrobenzene (2)**

To a solution of 4-fluoronitrobenzene 1 (7.5 g, 0.0532 moles, 1.0 equiv.) in *N,N*-dimethylformamide (75 mL), sodium azide (5.2 g, 0.0797 moles, 1.5 equiv.) was added slowly at room temperature with continuous stirring. The reaction mixture was then heated to 100 °C and stirred at this temperature for 3h. The progress of the reaction was monitored by thin-layer chromatography (TLC). Upon completion, the reaction mixture was cooled to room temperature, followed by the addition of water (150 mL), leading to the precipitation of a solid product. The turbid reaction mass was filtered, and the obtained solid was washed with water (25 mL). The product was then dried under reduced pressure, gave a yellow solid 1-azido-4-nitrobenzene 2 (8.291 g, 96.0% yield).

**Synthesis of intermediate ethyl 1-(4-nitrophenyl)-1*H*-1,2,3-triazole-4-carboxylate (4)**

To a solution of 1-azido-4-nitrobenzene 2 (8.0 g, 0.0487 moles, 1.0 equiv.) in a mixture of ethanol and water (7:3, 80 mL), ethyl propiolate 3 (5.4 mL, 0.0536 moles, 1.1 equiv.), sodium ascorbate (1.93 g, 0.0098 moles, 0.2 equiv.), and copper(II) sulfate pentahydrate (0.61 g, 0.0024 moles, 0.05 equiv.) were added gradually at room temperature with continuous stirring. The reaction mixture was subsequently heated to 80 °C and stirred at 80 °C for 2h. The progress of the reaction was monitored using thin-layer chromatography (TLC). Upon completion of the reaction, the mixture was filtered through a celite bed to remove iron oxide. The filtrate was evaporated, and the resultant reaction mass was extracted using ethyl acetate (3 x 40 mL). The organic layer was separated, combined, dried over anhydrous sodium sulfate, and evaporated under reduced pressure to afford a crude compound. This compound was further purified by crystallization in ethanol, resulting in a yellow amorphous solid ethyl 1-(4-nitrophenyl)-1H-1,2,3-triazole-4-carboxylate 4 (11.60 g, 91.0% yield).

**Synthesis of intermediate ethyl 1-(4-aminophenyl)-1*H*-1,2,3-triazole-4-carboxylate (5)**

To a solution of ethyl 1-(4-nitrophenyl)-1*H*-1,2,3-triazole-4-carboxylate 4 (5.0 g, 0.01907 moles, 1.0 eq.) in ethanol (100 mL) was charged 10% w/w palladium on charcoal (100 mg, 50% wet basis) under nitrogen gas atmosphere. Then, the reactor was further flushed with nitrogen, followed by hydrogen gas. The reactor was further filled with hydrogen gas with pressure 5.0 psi. Reaction mass was heated to 75-80 °C and continued at 75-80 °C temperature for 6h. After completion of the reaction, the reaction mixture was cooled to room temperature and filtered through a celite bed. Filtrate was partially evaporated under reduced pressure at 40-45 °C. The resultant suspension was cooled to room temperature and stirred at room temperature for 3 h, then filtered to remove the solid mass and washed with cold ethanol. The resultant off-white solid compound dried under reduced pressure gave ethyl 1-(4-aminophenyl)-1H-1,2,3-triazole-4-carboxylate 5 (3.94 g, 89% yield).

**General procedure for synthesis of derivatives (7a-h)**

To a solution of ethyl 1-(4-aminophenyl)-1*H*-1,2,3-triazole-4-carboxylate **5** (1.0 mmol) in *N,N*-dimethylformamide (5.0 mL), triethylamine (1.5 mmol) was added. The reaction mixture was then cooled to 0 °C, and the corresponding sulfonyl chloride (1.1 mmol) was added dropwise at 0 °C. The reaction mixture was subsequently warmed to room temperature and stirred continuously for 2h. The progress of the reaction was monitored by thin-layer chromatography (TLC). Upon completion, the reaction was quenched with ice-cold water (20 mL) and extracted with ethyl acetate (3 x 30 mL). The combined organic layers were washed with brine (20 mL), dried over anhydrous sodium sulphate, and then evaporated under reduced pressure. The resulting crude material was purified by column chromatography using silica gel and 2-6% methanol in dichloromethane as the eluent, yielding the desired solid product (7a-h, of 83-94%).

**Spectroscopic data of derivatives (7a-j)**

**Ethyl 1-(4-((4-methylphenyl)sulfonamido)phenyl)-1*H*-1,2,3-triazole-4-carboxylate (7a)**

Yield: 92% (356 mg); Off white solid, mp 204-206 °C; IR (KBr) 3149, 3065, 1723 cm^−1^; ^1^H-NMR (400 MHz, DMSO-d_6_) δ 1.32 (t, *J* = 7.2 Hz, 3H), 2.33 (s, 3H), 4.34 (q, *J* = 7.2 Hz, 2H), 7.28 (d, *J* = 9.0 Hz, 2H), 7.37 (d, *J* = 8.0 Hz, 2H), 7.70 (d, *J* = 8.0 Hz, 2H), 7.83 (d, *J* = 9.0 Hz, 2H), 9.33 (s, 1H), 10.62 (s, 1H); ^1^H-NMR (400 MHz, DMSO-d_6_ with D_2_O) δ 1.30 (t, *J* = 7.2 Hz, 3H), 2.30 (s, 3H), 4.32 (q, *J* = 7.2 Hz, 2H), 7.28 (d, *J* = 9.0 Hz, 2H), 7.35 (d, *J* = 8.4 Hz, 2H), 7.69 (d, *J* = 8.4 Hz, 2H), 7.81 (d, *J* = 9.0 Hz, 2H), 9.28 (s, 1H); ^13^C-NMR (100 MHz, DMSO-d_6_): δ 14.16, 20.94, 60.74, 120.15, 121.72, 126.72, 126.98, 129.84, 131.86, 136.37, 138.68, 139.64, 143.58, 160.09; HRMS (ESI): calculated for C_18_H_19_N_4_O_4_S, 387.1122 (MH^+^); found: m/z, 387.1137.

**Ethyl 1-(4-((3-methylphenyl)sulfonamido)phenyl)-1*H*-1,2,3-triazole-4-carboxylate (7b)**

Yield: 84% (325 mg); Off white solid, mp 195-197 °C; IR (KBr) 3132, 3072, 1723 cm^−1^; ^1^H-NMR (400 MHz, DMSO-d_6_) δ 1.32 (t, *J* = 7.2 Hz, 3H), 2.35 (s, 3H), 4.34 (q, *J* = 7.2 Hz, 2H), 7.29 (dd, *J* = 7.0, 2.1 Hz, 2H), 7.44-7.46 (m, 2H), 7.60-7.65 (m, 2H), 7.84 (dd, *J* = 7.0, 2.1 Hz, 2H), 9.34 (s, 1H), 10.67 (s, 1H); ^13^C-NMR (100 MHz, DMSO-d_6_): δ 14.17, 20.81, 60.74, 120.17, 121.73, 123.83, 126.84, 126.99, 129.25, 129.27, 131.89, 133.81, 138.61, 139.17, 139.23, 139.65, 160.10; HRMS (ESI): calculated for C_18_H_19_N_4_O_4_S, 387.1122 (MH^+^); found: m/z, 387.1144.

**Ethyl 1-(4-((4-fluorophenyl)sulfonamido)phenyl)-1*H*-1,2,3-triazole-4-carboxylate (7c)**

Yield: 90% (351 mg); Off white solid, mp 194-196 °C; IR (KBr) 3250, 3137, 1709 cm^−1^; ^1^H-NMR (400 MHz, DMSO-d_6_) δ 1.32 (t, *J* = 7.2 Hz, 4H), 4.34 (q, *J* = 7.2 Hz, 2H), 7.29 (d, *J* = 8.8 Hz, 2H), 7.42 (t, *J* = 8.8 Hz, 2H), 7.85-7.89 (m, 4H), 9.35 (s, 1H), 10.72 (s, 1H); ^13^C-NMR (100 MHz, DMSO-d_6_): δ 14.16, 60.75, 116.55, 116.78, 120.60, 121.75, 127.01, 129.75, 129.84, 132.16, 135.55, 138.34, 139.69, 160.09, 163.19, 165.69; HRMS (ESI): calculated for C_17_H_16_FN_4_O_4_S, 391.0871 (MH^+^); found: 391.0891.

**Ethyl 1-(4-((3-fluorophenyl)sulfonamido)phenyl)-1*H*-1,2,3-triazole-4-carboxylate (7d)**

Yield: 83% (324 mg); Off white solid, mp 203-205 °C; IR (KBr) 3167, 2878, 1720 cm^−1^; ^1^H-NMR (400 MHz, DMSO-d_6_) δ 1.32 (t, *J* = 7.2 Hz, 3H), 4.34 (q, *J* = 7.2 Hz, 2H), 7.31 (d, *J* = 8.8 Hz, 2H), 7.50-7.55 (m, 1H), 7.62-7.66 (m, 3H), 7.87 (d, *J* = 9.0 Hz, 2H), 9.36 (s, 1H), 10.82 (s, 1H); ^13^C-NMR (100 MHz, DMSO-d_6_): δ 14.18, 60.78, 113.63, 113.87, 120.37, 120.59, 120.70, 121.80, 123.00, 127.05, 131.90, 131.98, 132.28, 138.11, 139.69, 141.16, 141.23, 160.10, 160.45, 162.92; HRMS (ESI): calculated for C_17_H_16_FN_4_O_4_S, 391.0871 (MH^+^); found: m/z, 391.0892.

**Ethyl 1-(4-((4-chlorophenyl)sulfonamido)phenyl)-1*H*-1,2,3-triazole-4-carboxylate (7e)**

Yield: 94% (383 mg); Off white solid, mp 198-200 °C; IR (KBr) 3152, 3068, 1721 cm^−1^; ^1^H-NMR (400 MHz, DMSO-d_6_) δ 1.32 (t, *J* = 7.2 Hz, 3H), 4.34 (d, *J* = 7.2 Hz, 2H), 7.29 (d, *J* = 8.8 Hz, 2H), 7.66 (d, *J* = 8.6 Hz, 2H), 7.81 (d, *J* = 8.6 Hz, 2H), 7.87 (d, *J* = 8.8 Hz, 2H), 9.36 (s, 1H), 10.78 (s, 1H); ^13^C-NMR (100 MHz, DMSO-d_6_): δ 14.19, 60.79, 120.70, 121.78, 127.04, 128.65, 129.64, 132.25, 138.02, 138.12, 138.20, 139.70, 160.11; HRMS (ESI): calculated for C_17_H_16_ClN_4_O_4_S, 407.0575 (MH^+^); found: m/z, 407.0596.

**Ethyl 1-(4-((3-chlorophenyl)sulfonamido)phenyl)-1*H*-1,2,3-triazole-4-carboxylate (7f)**

Yield: 87% (354 mg); Off white solid, mp 156-158 °C; IR (KBr) 3147, 3071, 1723 cm^−1^; ^1^H-NMR (400 MHz, DMSO-d_6_) δ 1.32 (t, *J* = 7.2 Hz, 3H), 4.34 (q, *J* = 7.2 Hz, 2H), 7.30 (dd, *J* = 6.8, 2.0 Hz, 2H), 7.62 (d, *J* = 8.1 Hz, 1H), 7.72-7.77 (m, 2H), 7.83 (t, *J* = 1.8 Hz, 1H), 7.88 (dd, *J* = 7.1, 2.0 Hz, 2H), 9.37 (s, 1H), 10.83 (s, 1H); ^13^C-NMR (100 MHz, DMSO-d_6_): δ 14.19, 60.79, 120.75, 121.83, 125.45, 126.25, 127.07, 131.58, 132.34, 133.27, 134.02, 138.05, 139.70, 141.02, 160.11; HRMS (ESI): calculated for C_17_H_16_ClN_4_O_4_S, 407.0575 (MH^+^); found: m/z, 407.0596.

**Ethyl 1-(4-((4-methoxyphenyl)sulfonamido)phenyl)-1*H*-1,2,3-triazole-4-carboxylate (7g)**

Yield: 89% (358 mg); Off white solid, mp 175-177 °C; IR (KBr) 3145, 3070, 1720 cm^−1^; ^1^H-NMR (400 MHz, DMSO-d_6_) δ 1.32 (t, *J* = 7.2 Hz, 3H), 3.79 (s, 3H), 4.34 (q, *J* = 7.3 Hz, 2H), 7.08 (d, *J* = 8.8 Hz, 2H), 7.28 (d, *J* = 8.8 Hz, 2H), 7.75 (d, *J* = 9.2 Hz, 2H), 7.84 (d, *J* = 8.8 Hz, 2H), 9.34 (s, 1H), 10.56 (s, 1H); ^13^C-NMR (100 MHz, DMSO-d_6_): δ 14.16, 55.64, 60.75, 114.53, 120.09, 121.68, 126.96, 128.94, 130.55, 130.80, 132.91, 138.81, 139.66, 162.61; HRMS (ESI): calculated for C_18_H_19_N_4_O_5_S, 403.1071 (MH^+^); found: m/z, 403.1075.

**Ethyl 1-(4-((3,4-dichlorophenyl)sulfonamido)phenyl)-1*H*-1,2,3-triazole-4-carboxylate (7h)**

Yield: 88% (388 mg); Off white solid, mp 234-236 °C; IR (KBr) 3164, 3131, 1725 cm^−1^; ^1^H-NMR (400 MHz, DMSO-d_6_) δ 1.32 (t, *J* = 7.2 Hz, 3H), 4.34 (t, *J* = 7.2 Hz, 2H), 7.31 (d, *J* = 8.8 Hz, 2H), 7.73 (dd, *J* = 8.4, 1.6 Hz, 1H), 7.88 (t, *J* = 8.4 Hz, 3H), 8.01 (d, *J* = 1.6 Hz, 1H), 9.37 (s, 1H), 10.84 (s, 1H); ^13^C-NMR (100 MHz, DMSO-d_6_): δ 14.16, 60.75, 121.04, 121.83, 126.78, 127.04, 128.41, 131.90, 132.34, 132.50, 136.37, 137.78, 139.40, 139.70, 160.07; C_17_H_15_Cl_2_N_4_O_4_S, 441.0186 (MH^+^); found: m/z, 441.0205.

**Ethyl 1-(4-((2,5-dichlorophenyl)sulfonamido)phenyl)-1*H*-1,2,3-triazole-4-carboxylate (7i)**

Yield: 84 % (370 mg); Off white solid, mp 243-245 °C; IR (KBr) 3309, 3130, 1724 cm^−1^;^1^H-NMR (400 MHz, DMSO-d_6_) δ 1.32 (t, *J* = 7.2 Hz, 3H), 4.34 (d, *J* = 7.2 Hz, 2H), 7.31 (d, *J* = 9.0 Hz, 2H), 7.69-7.75 (m, 2H), 7.87 (d, *J* = 9.0 Hz, 2H), 8.08 (d, *J* = 2.4 Hz, 1H), 9.34 (s, 1H), 11.20 (s, 1H); ^13^C-NMR (100 MHz, DMSO-d_6_): δ 14.17, 60.76, 119.88, 121.92, 127.06, 129.55, 130.85, 132.14, 132.34, 133.78, 134.74, 137.44, 137.73, 139.66, 160.09; HRMS (ESI): calculated for C_17_H_15_Cl_2_N_4_O_4_S, 441.0186 (MH^+^); found: m/z, 441.0210.

**Ethyl 1-(4-((3,5-dichlorophenyl)sulfonamido)phenyl)-1*H*-1,2,3-triazole-4-carboxylate (7j)**

Yield: 87% (383 mg); Off white solid, mp 249-251 °C; IR (KBr) 3394, 3116, 1726 cm^−1^; ^1^H-NMR (400 MHz, DMSO-d_6_) δ 1.32 (t, *J* = 7.2 Hz, 3H), 4.34 (t, *J* = 7.2 Hz, 2H), 7.50 (d, *J* = 2.0 Hz, 2H), 7.64 (d, *J* = 9.2 Hz, 2H), 7.79 (d, *J* = 1.6 Hz, 1H), 8.10 (d, *J* = 8.8 Hz, 2H), 9.50 (s, 1H), 11.04 (bs, 1H); ^13^C-NMR (100 MHz, DMSO-d_6_): δ 14.19, 60.85, 120.09, 121.11, 121.82, 124.25, 125.24, 128.06, 133.00, 133.62, 135.27, 142.22, 160.09; C_17_H_15_Cl_2_N_4_O_4_S, 441.0186 (MH^+^); found: m/z, 441.0196

**Biological Activity**

***Antibacterial Activity***: The synthesised 1,2,3-triazole derivatives were assessed for their antibacterial activity against a set of four bacterial strains. This set consisted of two gram-negative bacteria, *Escherichia coli* (MTCC 443) and *Pseudomonas aeruginosa* (MTCC 1688), and two gram-positive bacteria, *Staphylococcus aureus* (MTCC 96) and *Streptococcus pyogenes* (MTCC 442). The antibacterial activity was evaluated using the broth microdilution assay for determining minimum inhibitory concentration (MIC), following established microbiological protocols. Mueller–Hinton broth was used as growth media. DMSO was used as a diluent to get the desired drug concentration to test standard bacterial strains. Each synthesised drug was diluted to obtain a 2000 μg/ml concentration as a stock solution. In primary screening, 500, 250, and 125 μg/ml concentrations of the synthesised drugs were used. The active synthesised drugs found in this primary screening were further tested in a second set of dilutions against all microorganisms. The drugs found active in primary screening were similarly diluted to obtain 100, 50, 25, 12.5, and 6.25 μg/ml concentrations. The highest dilution showing at least 99 % inhibition is taken as the MIC.

***Antifungal Activity***: The antifungal activity of the synthesised 1,2,3-triazole derivatives were evaluated against three fungal strains: *Candida albicans* (MTCC 227), *Aspergillus niger* (MTCC 282), and *Aspergillus clavatus* (MTCC 1323). Sabouraud dextrose broth (for fungi) was used as growth media. The antifungal activity was evaluated following an analogous broth microdilution protocol to that used for antibacterial assays, with appropriate modifications for fungal strains.

***Antimalarial Activity*:** The antimalarial activity of the synthesised 1,2,3-triazole derivatives were evaluated against *Plasmodium* *falciparum* (strain 3D7). The *in* *vitro* antimalarial activity was determined using the standard parasite growth inhibition assay, based on the SYBR Green I fluorescence method. A dose–response assay was performed using serial dilutions of the test compounds to evaluate their inhibitory effect on parasite growth. All experiments were performed in triplicate (n = 3), and results are expressed as the mean of IC50 values of parasite growth.

***Antitubercular Activity***: The antitubercular efficacy of triazole derivatives were evaluated against *Mycobacterium tuberculosis H37Rv* using the Löwenstein-Jensen (L.J.) medium method. The test compounds were incorporated into sterile L.J. medium at the desired concentrations, such as 500, 100, 50, 25, 12.5, and 6.25 μg/ml. The medium was inoculated with a standardised suspension of Mycobacterium tuberculosis and incubated at 37°C. Growth was monitored periodically for up to 6–8 weeks, and the inhibitory effect of the test compounds was determined by comparing colony growth in treated and untreated control cultures. The minimum inhibitory concentration (MIC) was defined as the lowest concentration that completely inhibited visible mycobacterial growth.

**In silico study**

*With antibacterial target DNA gyrase*

*Protein Preparation:* To assess their effective antibacterial mechanism, these compounds were docked against the active site of the DNA gyrase of *Staphylococcus aureus* (PDB ID: 7FVT, Resolution = 2.08 Å), which was retrieved from the RCSB Protein Data Bank (<https://www.rcsb.org/structure/7FVT>).^1^ Protein structures were prepared using the Protein Preparation Wizard in Maestro (Version 13.0, Schrodinger, LLC, New York, NY, 2021, 2024). The preparation involved assigning bond orders, adding hydrogen atoms, creating disulfide bonds, and deleting waters beyond 5.0 Å from het groups.^2^ Missing side chains and loops were filled using Prime. The protonation states of residues were assigned using Epik at pH 7.4 ± 2.0. Hydrogen bonding networks were optimised, and restrained minimisation was performed using the OPLS4 force field, converging heavy atoms to an RMSD of 0.30 Å.^3^

*Molecular Docking:* The 3D structures of triazole compounds were prepared and neutralised using LigPrep and subjected to a molecular docking analysis with the refined 3D structure of the DNA gyrase protein using the XP scoring function in the Glide interface of Schrödinger Suites (Glide 13.0, Schrodinger, LLC, New York, NY, 2021, 2024). A cuboidal docking grid was constructed based on the co-crystallized ligand,

**Figure SI 1.** Two-dimensional molecular docking interaction diagrams of the synthesized compounds **(a) 8b, (b) 8c, (c) 8d, (d) 8e, (e) 8h, and (f) 8i** within the active site of **DNA gyrase**, illustrating the key ligand–residue interactions involved in their predicted binding.

which was critically bound to the active site of the protein and the embedded DNA fragment. During the grid generation, the van der Waals scaling factor was set to 1.0 for the receptor with a partial charge cutoff of 0.25, indicating that the receptor was treated as rigid and sterically defined. The docking grid has dimensions of 39.67 × 39.67 × 39.67 Å³ along the X, Y, and Z axes, respectively. Validation of the docking results was done by means of superimposing the docked sunitinib ligand over the corresponding LigPrep energy-minimised sunitinib with the help of the structure alignment tool. The analysis gave RMSD values of 0.9109 Å for the co-crystallized ligand, which were considered permissible for confirmation of docking results.^4^

**Figure SI 2.** 2D molecular docking interaction diagrams of the synthesized compounds **(a) co-crystallised ligand, (b) cephalexin, (c) amoxicillin, and (d) cefixime** within the active site of **DNA gyrase**. (e) Colour-coded legend describing the residue types and interaction categories represented in the docking diagrams.

*With antifungal target lanosterol 14α-demethylase (CYP51)*

*Protein Preparation:* To assess their effective antibacterial mechanism, these compounds were docked against the active site of the DNA gyrase of *Staphylococcus aureus* (PDB ID: 5V5Z, Resolution = 2.90 Å), which was retrieved from the RCSB Protein Data Bank (<https://www.rcsb.org/structure/5V5Z>).^5^ Protein structures were prepared using the Protein Preparation Wizard in Maestro (Version 13.0, Schrodinger, LLC, New York, NY, 2021, 2024). The preparation involved assigning bond orders, adding hydrogen atoms, creating disulfide bonds, and deleting waters beyond 5.0 Å from het groups.^2^ Missing side chains and loops were filled using Prime. The protonation states of residues were assigned using Epik at pH 7.4 ± 2.0. Hydrogen bonding networks were optimised, and restrained minimisation was performed using the OPLS4 force field, converging heavy atoms to an RMSD of 0.30 Å.^3^

**Figure SI 3.** 2D molecular docking interaction diagrams of the synthesized compounds **(a) 8a, (b) 8c, (c) 8d, (d) 8e, (e) 8h, and (f) 8i** within the active site of *lanosterol 14α-demethylase (CYP51)*, illustrating the key ligand-residue interactions involved in their predicted binding.

*Molecular Docking:* The 3D structures of triazole compounds were prepared and neutralised using LigPrep and subjected to a molecular docking analysis with the refined 3D structure of the DNA gyrase protein using the XP scoring function in the Glide interface of Schrödinger Suites (Glide 13.0, Schrodinger, LLC, New York, NY, 2021, 2024). A cuboidal docking grid was constructed based on the co-crystallized ligand, which was critically bound to the active site of the protein and the embedded DNA fragment. During the grid generation, the van der Waals scaling factor was set to 1.0 for the receptor with a partial charge cutoff of 0.25, indicating that the receptor was treated as rigid and sterically defined. The docking grid has dimensions of 38 × 38 × 38 Å³ along the X, Y, and Z axes, respectively. Validation of the docking results was done by means of superimposing the docked sunitinib ligand over the corresponding LigPrep energy-minimised sunitinib with the help of the structure alignment tool. The analysis gave RMSD values of 1.8865 Å for the co-crystallized ligand, which were considered permissible for confirmation of docking results.^4^

**Figure SI 4.** 2D molecular docking interaction diagrams of the synthesized compounds **(a) co-crystallised ligand, (b) Fluconazole, (c) Ketoconazole, and (e) Abafungin** within the active site of *lanosterol 14α-demethylase (CYP51)*. (d) Colour-coded legend describing the residue types and interaction categories represented in the docking diagrams.

*With c-KIT tyrosine kinase*

*Protein Preparation:* To assess their effective c-KIT tyrosine kinase inhibitor properties, these compounds were docked against the ATP-binding site of the c-KIT kinase domain of the crystal structure of human kinase protein, which was retrieved from the RCSB Protein Data Bank (PDB ID: 1T46, Resolution: 1.60 Å) (<https://www.rcsb.org/structure/1T46>).^6^ Protein structures were prepared using the Protein Preparation Wizard in Maestro (Version 13.0, Schrodinger, LLC, New York, NY, 2021, 2024). The preparation involved assigning bond orders, adding hydrogen atoms, creating disulfide bonds, and deleting waters beyond 5.0 Å from het groups.^2^ Missing side chains and loops were filled using Prime. The protonation states of residues were assigned using Epik at pH 7.4 ± 2.0. Hydrogen bonding networks were optimised, and restrained minimisation was performed using the OPLS4 force field, converging heavy atoms to an RMSD of 0.30 Å.^3^

*Molecular Docking:* The 3D structures of triazole compounds were prepared and neutralised using LigPrep and subjected to a molecular docking analysis with the refined 3D structure of c-KIT tyrosine kinase protein using the XP scoring function in the Glide interface of Schrödinger Suites (Glide 13.0, Schrodinger, LLC, New York, NY, 2021, 2024). A cuboidal docking grid was constructed based on the specific orientation of the ATP binding site, targeting GLU671, CYS673, ASP677, and ARG796, which are known to play critical roles in ligand recognition and binding. During the grid generation, the van der Waals scaling factor was set to 1.0 for the receptor with a partial charge cutoff of 0.25, indicating that the receptor was treated as rigid and sterically defined. The docking grid has dimensions of 33.4 × 25.14 × 42.00 Å³ along the X, Y, and Z axes, respectively. Validation of the docking results was done by means of superimposing the docked sunitinib ligand over the corresponding LigPrep energy-minimised sunitinib with the help of the structure alignment tool. The analysis gave RMSD values of 1.4242 Å for the ATP binding site, which were considered permissible for confirmation of docking results.^4^

**Figure SI5.** Interaction fingerprint of triazole ligands with c-KIT tyrosine kinase

*MM-GBSA (Molecular Mechanics Generalised Born Surface Area):* The MM-GBSA method for the study of protein-ligand interactions and estimating the binding free energies (ΔG_bind_) in computational chemistry and structural biology is very popular and effective. It combines energies from molecular mechanics with solvation models to provide information related to the stability and strength of protein-ligand interactions.^7^ As part of the analysis, MM/GBSA calculations were performed on recognised hits obtained from docking studies using the Prime module within the Schrödinger Suite (Prime 13.0; Schrodinger, LLC, New York, NY, 2021, 2024). For the estimation of the energy, the Volume Surface Generalised Born (VSGB 2.0) model was employed, which includes an optimised implicit solvent model with physics-based corrections for hydrogen bonding, π–π interactions, self-contact interactions, and hydrophobic effects.^8^ The OPLS5 force field was used during this process to minimise the energy of the structures.

**Table SI1. mmGBSA-Study of ligands with c-KIT tyrosine kinase**

| **Ligands** | **ΔG_bind_** | **ΔG_Coulomb_** | **ΔG_Covalent_** | **ΔG_Hbond_** | **ΔG_Lipo_** | **ΔG_Packing_** | **ΔG_Solv-GB_** | **ΔG_vdW_** | **Lig_Strain**  **Energy** |
| --- | --- | --- | --- | --- | --- | --- | --- | --- | --- |
| **8e** | **-73.695** | **23.901** | **8.022** | **-0.872** | **-33.198** | **-0.938** | **-14.122** | **-56.490** | **11.800** |
| **8c** | **-70.367** | **31.879** | **1.085** | **-0.865** | **-27.825** | **-0.951** | **-18.469** | **-55.222** | **5.377** |
| **8a** | **-77.070** | **25.877** | **5.489** | **-0.855** | **-32.643** | **-0.901** | **-17.080** | **-56.957** | **5.971** |
| 8g | -77.901 | 35.741 | -0.143 | -0.773 | -33.733 | -0.881 | -19.263 | -58.849 | 4.380 |
| 8h | -86.708 | 28.871 | 3.071 | -0.858 | -39.447 | -0.864 | -19.829 | -57.652 | 10.104 |
| 8b | -69.428 | 24.235 | 7.550 | -0.806 | -32.802 | -0.914 | -14.013 | -52.680 | 8.065 |
| 8f | -75.794 | 24.489 | 4.971 | -0.753 | -36.021 | -0.928 | -14.165 | -53.387 | 5.834 |
| 8d | -67.314 | 17.764 | 8.458 | -1.116 | -29.373 | -0.202 | -17.076 | -45.771 | 9.832 |
| 8i | -55.135 | 10.383 | 7.278 | -0.728 | -24.518 | -0.057 | 2.491 | -49.985 | 11.832 |
| 8j | -70.846 | 23.164 | 2.870 | -1.197 | -41.161 | 0.000 | -9.338 | -45.184 | 4.065 |
| Dasatinib | -100.857 | -20.475 | 5.592 | -0.586 | -52.586 | -0.378 | 25.790 | -58.215 | 5.995 |
| Motesanib | -85.732 | -10.699 | 2.175 | -1.278 | -49.660 | -0.005 | 27.958 | -54.223 | 12.615 |
| Sunitinib | -83.730 | -20.559 | 7.162 | -0.619 | -57.510 | -0.108 | 25.421 | -37.517 | 11.541 |

*ADME and Toxicity Study:* When assessing drug-likeness, drug candidates ought to be evaluated for pharmacokinetic properties like ADME (Absorption, Distribution, Metabolism, and Excretion).^9^ The ADME properties of the identified molecules were then assessed in QikProp 13.0 from Schrödinger, LLC (New York, NY, 2021, 2024). QikProp calculates several physicochemical descriptors, which can be used to predict ADME properties and which contribute to an overall indication of drug likeness given by the "stars" parameter, indicating the number of descriptors that lie outside the specified optimal range for 95% of known drugs. The "stars" range from 0 to 5, with 0 having maximum drug-likeness and 5 having minimum drug-likeness. Key factors such as molecular weight, hydrogen bond donors and acceptors, aqueous solubility, the octanol/water partition coefficient, and solvent-accessible surface area were also some of the assessed parameters.

*Molecular Dynamics:* To further validate the docking and MM-GBSA findings, molecular dynamics (MD) simulations were carried out using the GROMACS software package with the CHARMM36 all-atom force field.^10-12^ Ligand topology files were generated through SwissParam before simulation.^13^ A cubic simulation box was constructed and solvated using the TIP3P water model.^14^ The system underwent energy minimisation, followed by equilibration under constant volume (NVT) and constant pressure (NPT) conditions for 100 ps each. Subsequently, a 50 ns production MD run was performed to analyse structural stability and dynamics through parameters such as root mean square deviation (RMSD), root mean square fluctuation (RMSF), and radius of gyration (Rg).

**References**

1. C.D. Mol, D.R. Dougan, T.R. Schneider, R.J. Skene, M.L. Kraus, D.N. Scheibe, G.P. Snell, H. Zou, B.C. Sang, K.P. Wilson, *Journal of Biological Chemistry*, 2004, *279*(30), 31655-31663.
2. M. Kumari, B. Rathi, G. Singh, Journal of Integrated Science and Technology 2025, 13, 1074-1074.
3. M. Mallick, T. R. Yoithap Prabhunath, S. Kumari, M. E. Sobhia, Journal of Biomolecular Structure and Dynamics 2024, 42, 13768-13778.
4. D. Ramírez, J. Caballero, in *Molecules, Vol. 23*, **2018**.
5. J.G. Cumming, L. Kreis, H. Kühne, R. Wermuth, M. Vercruysse, C. Kramer, M.G. Rudolph, Z. Xu, *ACS Medicinal Chemistry Letters*, **2023**, *14*(7), 993-998.
6. M.V. Keniya, M. Sabherwal, R.K. Wilson, M.A. Woods, A.A. Sagatova, J.D. Tyndalland, B.C. Monk, *Antimicrobial agents and chemotherapy*, **2018**, *62*(11),10-1128.
7. S. Genheden, U. Ryde, *Expert Opinion on Drug Discovery* **2015**, *10*, 449-461.
8. J. Li, R. Abel, K. Zhu, Y. Cao, S. Zhao, R. A. Friesner, *Scientific Reports* **2023**, *13*, 3743.
9. G. Lanka, D. Begum, S. Banerjee, N. Adhikari, P. Yogeeswari, B. Ghosh, *Computers in Biology and Medicine* **2023**, *166*, 107481.
10. S. Pronk, S. Páll, R. Schulz, P. Larsson, P. Bjelkmar, R. Apostolov, M. R. Shirts, J. C. Smith, P. M. Kasson, D. Van Der Spoel, *Bioinformatics* **2013**, *29*, 845-854;
11. S. Páll, M. J. Abraham, C. Kutzner, B. Hess, E. Lindahl, Springer, pp. 3-27;
12. B. R. Brooks, C. L. Brooks Iii, A. D. Mackerell, L. Nilsson, R. J. Petrella, B. Roux, Y. Won, G. Archontis, C. Bartels, S. Boresch, *J. Comput. Chem* **2009**, *30*, 1545-1614.
13. V. Zoete, M. A. Cuendet, A. Grosdidier, O. Michielin, *Journal of computational chemistry* **2011**, *32*, 2359-2368.
14. W. L. Jorgensen, J. Chandrasekhar, J. D. Madura, R. W. Impey, M. L. Klein, *The Journal of Chemical Physics* **1983**, *79*, 926-935.

**^1^H and ^13^C NMR Spectra**

**^1^H and ^13^C NMR Spectra of 7g and 7j** (Spectra of the rest of the compounds of this series were available in the article “Design, Synthesis, and Biological Evaluation of Novel Sulfonamide-Linked 1,2,3-Triazole Conjugates, Discover Chemistry, 2026, DOI: )

**Ethyl 1-(4-((4-methoxyphenyl)sulfonamido)phenyl)-1*H*-1,2,3-triazole-4-carboxylate (7g)**

**Ethyl 1-(4-((3,5-dichlorophenyl)sulfonamido)phenyl)-1*H*-1,2,3-triazole-4-carboxylate (7j)**

**^1^H and ^13^C NMR Spectra of (8a-j)**

**8a**. ***N*-hydroxy-1-(4-((4-methylphenyl)sulfonamido)phenyl)-1*H*-1,2,3-triazole-4-carboxamide**

**8b. *N*-hydroxy-1-(4-((3-methylphenyl)sulfonamido)phenyl)-1*H*-1,2,3-triazole-4-carboxamide**

**8c. 1-(4-((4-fluorophenyl)sulfonamido)phenyl)-*N*-hydroxy-1*H*-1,2,3-triazole-4-carboxamide**

**8d. 1-(4-((3-fluorophenyl)sulfonamido)phenyl)-*N*-hydroxy-1*H*-1,2,3-triazole-4-carboxamide**

**8e. 1-(4-((4-chlorophenyl)sulfonamido)phenyl)-*N*-hydroxy-1*H*-1,2,3-triazole-4-carboxamide**

**8f. 1-(4-((3-chlorophenyl)sulfonamido)phenyl)-*N*-hydroxy-1*H*-1,2,3-triazole-4-carboxamide**

**8g. *N*-hydroxy-1-(4-((3-methoxyphenyl)sulfonamido)phenyl)-1*H*-1,2,3-triazole-4-carboxamide**

**8h. 1-(4-((3,4-dichlorophenyl)sulfonamido)phenyl)-*N*-hydroxy-1*H*-1,2,3-triazole-4-carboxamide**

**8i. 1-(4-((2,5-dichlorophenyl)sulfonamido)phenyl)-*N*-hydroxy-1*H*-1,2,3-triazole-4-carboxamide**

**8j. 1-(4-((3,5-dichlorophenyl)sulfonamido)phenyl)-*N*-hydroxy-1*H*-1,2,3-triazole-4-carboxamide**
